# Assessment of Multipollutant Exposures During Pregnancy Using Silicone Wristbands

**DOI:** 10.3389/fpubh.2020.547239

**Published:** 2020-09-29

**Authors:** Brett T. Doherty, John L. Pearce, Kim A. Anderson, Margaret R. Karagas, Megan E. Romano

**Affiliations:** ^1^Department of Epidemiology, Geisel School of Medicine at Dartmouth, Hanover, NH, United States; ^2^Department of Public Health Sciences, Medical University of South Carolina, Charleston, SC, United States; ^3^Department of Environmental and Molecular Toxicology, Oregon State University, Corvallis, OR, United States

**Keywords:** wristband samplers, passive monitors, personal exposure, pollutant, exposome, mixture, pregnancy, self-organizing map

## Abstract

Silicone wristbands can assess multipollutant exposures in a non-invasive and minimally burdensome manner, which may be suitable for use among pregnant women. We investigated silicone wristbands as passive environmental samplers in the New Hampshire Birth Cohort Study, a prospective pregnancy cohort. We used wristbands to assess exposure to a broad range of organic chemicals, identified multipollutant exposure profiles using self-organizing maps (SOMs), and assessed temporal consistency and determinants of exposures during pregnancy. Participants (*n* = 255) wore wristbands for 1 week at 12 gestational weeks. Of 1,530 chemicals assayed, 199 were detected in at least one wristband and 16 were detected in >60% of wristbands. A median of 23 (range: 12,37) chemicals were detected in each wristband, and chemicals in commerce and personal care products were most frequently detected. A subset of participants (*n*=20) wore a second wristband at 24 gestational weeks, and concentrations of frequently detected chemicals were moderately correlated between time points (median intraclass correlation: 0.22; range: 0.00,0.69). Women with higher educational attainment had fewer chemicals detected in their wristbands and the total number of chemicals detected varied seasonally. Triphenyl phosphate concentrations were positively associated with nail polish use, and benzophenone concentrations were highest in summer. No clear associations were observed with other *a priori* relations, including certain behaviors, season, and socioeconomic factors. SOM analyses revealed 12 profiles, ranging from 2 to 149 participants, captured multipollutant exposure profiles observed in this cohort. The most common profile (*n* = 149) indicated that 58% of participants experienced relatively low exposures to frequently detected chemicals. Less common (*n* ≥ 10) and rare (*n* < 10) profiles were characterized by low to moderate exposures to most chemicals and very high and/or very low exposure to a subset of chemicals. Certain covariates varied across SOM profile membership; for example, relative to women in the most common profile who had low exposures to most chemicals, women in the profile with elevated exposure to galaxolide and benzyl benzoate were younger, more likely to be single, and more likely to report nail polish use. Our study illustrates the utility of silicone wristbands for measurement of multipollutant exposures in sensitive populations, including pregnant women.

## Introduction

Available evidence indicates that pregnant women in the United States are exposed to many environmental pollutants, including exposures from personal care products, consumer goods, indoor and outdoor air pollution, and dietary sources (1–3). The prenatal period is a uniquely sensitive period of development (4, 5), and chemical exposures can perturb maternal physiology and subsequently affect fetal developmental with long term effects for both mother and offspring (6, 7). Additionally, some chemicals may cross the placental barrier and directly expose the fetus to potentially toxic chemicals (8–10). Of particular concern are adverse impacts of exposures to multipollutant mixtures, as chemical exposures may act jointly, modify the effects of one another, and/or confound relations of one another (11–14). However, the assessment of multipollutant exposures among pregnant women is challenging because it is especially important to minimize risks and burdens of exposure assessments among this sensitive population.

Silicone wristbands are a promising passive monitoring technology, and their use among sensitive populations, including pregnant women, is supported by their non-invasive and minimally disruptive nature (15–19). Individuals wear the silicone wristbands for an established period (e.g., 1 week), during which time a wide variety of chemical pollutants are absorbed by the silicone during their typical activities; the chemicals are then extracted from the wristbands and their concentrations are quantified. Over 1,500 chemicals can be measured in a single wristband, providing insight into participants' environmental exposures to both individual chemical pollutants and multipollutant mixtures (20). Such broad exposure assessments enable the identification of prevalent pollutants and multipollutant mixtures, which may be prioritized for targeted research or intervention.

This emerging technology has been applied in several settings (15), including the assessment of exposures to flame retardants among maternal-child pairs (21) and school children (18) and the assessment of exposures to pesticides among agricultural workers (22) and non-occupationally exposed persons (23). However, there is limited evidence characterizing their use among pregnant populations; to date, a single study has used the wristbands to assess exposures to PAHs among pregnant women (16) and no studies have reported on their use to assess multipollutant exposures of many chemical classes.

Previous studies have indicated that pollutant exposures among pregnant women may be influenced by sociodemographic, behavioral, and anthropometric factors (24–27). The identification of predictors of pollutant exposures can identify vulnerable populations and support efforts to reduce harmful exposures during the sensitive prenatal period. The assessment of temporal consistency of pollutant exposures during pregnancy is also important to understand as pregnancy is a period of physiological and behavioral changes (e.g., physical activity, diet) and pollutant exposures may vary throughout pregnancy and may subsequently have different impacts on maternal health and fetal development. The characterization of exposures at different points may therefore enable identification of sensitive periods of exposure (4, 5). Additionally, it is important to assess the consistency of pollutant exposures measured by the wristbands at multiple periods of pregnancy to determine the utility of the wristbands to assess longer-term exposure to certain pollutants.

Therefore, we used silicone wristbands to measure and assess exposures to chemical pollutants in a cohort of pregnant women receiving prenatal care in New Hampshire. Specifically, we sought to identify common chemical exposures and chemical exposure patterns, identify potential predictors of these exposures, and assess temporal consistency of exposures during pregnancy characterized by the silicone wristband.

## Materials and Methods

### Study Sample

Data used in this analysis were collected as part of the New Hampshire Birth Cohort Study (NHBCS), an ongoing prospective cohort study initially conceived to study the effects of drinking water contaminants on pregnancy (especially arsenic and metal exposures among private well users). Eligible women were identified at participating prenatal care clinics in New Hampshire. Women were invited to participate in NHBCS if they satisfied the following eligibility criteria: 18–45 years of age, literate in English, used a private water system as the primary source of water at their residence, and resided at the same address since their last menstrual period and intended to remain at that residence throughout the pregnancy. Participants were prospectively followed throughout pregnancy, including two in-person visits at ~12 and 24 gestational weeks. Participants completed questionnaires that solicited information on a variety of demographic and lifestyle characteristics. NHBCS also extracted relevant information about participants from the prenatal medical record. Participants included in this analysis were women enrolled between March 2017 and December 2018 who wore and returned silicone wristbands at ~12 gestational weeks (*n* = 255). A subset of these participants (*n* = 20) were consecutively invited to wear a second wristband at ~24 gestational weeks for a pilot study of temporal consistency of the wristband measurements during pregnancy. Study materials and protocols for NHBCS were approved by the Committee for the Protection of Human Subjects at Dartmouth College, and all participants provided written informed consent.

### Assessment of Multipollutant Exposures With Silicone Wristbands

Exposure to a diverse suite of organic chemicals was assessed via silicone wristbands provided and analyzed by MyExposome, Inc. (Philadelphia, PA) (17, 19, 20). Prior to deployment, silicone wristbands (https://24hourwristbands.com, Houston, TX, USA) were cleaned and prepared using previously described methods (19). Briefly, wristbands were vacuum oven conditioned at 300°C for 12 h at 0.1 Torr (Vacuum Oven, Blue-M, model no. POM18VC, with Welch Duo-seal pump, model no. 1405). All participants in the study sample were provided with a silicone wristband during a study visit at ~12 gestational weeks, and a subset of these participants were provided a second wristband at ~24 gestational weeks. Silicone samplers were transferred to polytetrafluoroethylene (PTFE) bags before deployment. Participants had the option of receiving a regular sized or small sized wristband. Participants were instructed to wear the wristband on their right arm for 7 days and to wear the wristband while performing their normal activities, including sleeping and showering. Participants were instructed to avoid spreading any lotion directly onto the wristband and to record the time and date that they began wearing and finished wearing the wristbands. After wearing the wristbands for ~7 days, participants sealed the wristbands in PTFE bags and mailed them to the NHBCS study office. NHBCS received the wristbands and stored them at room temperature until they were mailed overnight to MyExposome, Inc. for analysis; chemical concentrations in the wristbands have been observed to remain stable in a variety of realistic temperature, storage, and transport scenarios (19).

Upon receipt at the MyExposome laboratory, wristbands were cleaned to remove particulate matter with two rinses of 18 Ω·cm and one of isopropanol (17). Prior to extraction, wristbands were stored in amber glass jars at −20°C. Deuterated analytes were added as recovery surrogates. Chemicals were extracted from the wristbands with two 50 mL volumes of ethyl acetate at ambient temperature. Sample extracts were combined and quantitatively reduced to one mL under nitrogen (Turbo-Vap L, Biotage, Charlotte, NC, USA; RapidVap, LabConco, Kansas City, MO, USA; N-EVAP 111, Organomation Associates, Berlin, MA, USA). Sample extracts were stored at 4°C prior to additional cleanup by solid phase extraction (SPE) (15, 17, 18). Sample aliquots of 100 uL underwent SPE using acetonitrile (Cleanert S C18, Agela Technologies, Torrance, CA, USA), were solvent exchanged to iso-octane (OA-SYS N-EVAP 111, Organomation Associates, Berlin, MA, USA), and stored at 4°C prior to instrument analysis.

The analytical screen of 1,530 chemicals was performed using a 6,890 N Gas Chromatograph (Agilent, Santa Clara, CA) with a 5975B Mass Selective Detector (Agilent, Santa Clara, CA) in full scan mode (a targeted analysis). Details about the quantitative method, including limits of quantitation, have been previously reported (20) and the full analyte list is available online (http://www.myexposome.com/fullscreen).

To ensure satisfaction of data quality objectives, quality control accounted for ~30% of the samples analyzed. Quality control samples included conditioning verification, trip blanks, laboratory processing blanks, post-deployment cleaning blanks, silicone dialysis blanks, SPE blanks, SPE duplicates, sample overspikes, instrument solvent blanks, and continuing calibration verifications. Sample background correction was conducted using the laboratory processing blanks. All duplicates and overspikes were within data quality objectives at 30–170% of the expected concentrations. All calibration verifications were within data quality objectives at ±30% of the true value for 70% of the target analytes (PAH method) or within 2.5 times the true value for 60% of the target analytes.

### Statistical Analyses

We calculated descriptive statistics of demographic characteristics and wristband utilization among the study sample. We calculated descriptive statistics of the chemical concentrations measured in the silicone wristbands, the total number of chemicals detected in each wristband, and among the following chemical categories provided by MyExposome, Inc. Chemical categories included chemicals in commerce (e.g., phthalates, organophosphate esters), personal care products (e.g., fragrances, UV-blockers), pesticides (e.g., permethrin, N,N-Diethyl-m-toluamide), flame retardants (e.g., organophosphate esters), polycyclic aromatic hydrocarbons (PAHs), consumer products (e.g., scents, food preservatives), and pharmacological (e.g., caffeine, butylated hydroxyanisole); some chemicals were assigned to multiple categories (Supplementary Table 1). (To note, chemicals may be used in applications beyond those identified by the MyExposome classifications; however, we use these classifications for simplicity and comparisons with other works). We calculated Spearman correlation coefficients among chemical concentrations measured in the silicone wristbands worn at 12 gestational weeks, limited to chemicals detected in >60% of wristbands.

To assess reproducibility of exposures, we calculated intraclass correlation coefficients (ICCs) between concentrations of the same chemical measured *in silicone* wristbands worn by participants at ~12 and 24 gestational weeks, limited to chemicals detected in >60% of wristbands at either time point. ICCs ≤ 0.4, between 0.4 and 0.75, and ≥0.75, respectively, designate poor, fair to good, and excellent reproducibility (28). We used a linear mixed effects model to obtain point estimates for the ICCs, where each chemical concentration was modeled with week of measurement as a fixed effect and participant as a random effect; we repeated these analyses in 1000 bootstrapped resamples to obtain 95% confidence intervals.

We used multivariable linear regression to identify potential predictors of the total number of chemicals detected in wristbands worn at 12 gestational weeks; variables included in the multivariable linear model included age (years, continuous), body mass index (kg/m^2^, continuous), educational attainment (less than college graduate, college graduate, any post-graduate), marital status (married, unmarried), race and ethnicity (White Non-Hispanic, other), parity (0, ≥1), self-reported smoke exposure during pregnancy (first- or second-hand exposure, none), gestational age the wristband was first worn (weeks, continuous), and season the wristband was worn (winter, spring, summer, fall). In these multivariable linear models, missing data were imputed using the multivariate imputation by chained equations via the *mice* package of R to generate 25 imputed datasets, and results were pooled across models (29).

We used multivariable linear regression to investigate *a priori* exposure-chemical relations, including the following: (1) reported nail polish use in the first trimester and total number of detected chemicals from personal care products and concentrations of triphenyl phosphate, di-n-butyl phthalate, diisobutyl phthalate, butyl benzyl phthalate, di-n-nonyl phthalate, and diethyl phthalate (30–34); (2) reported daily handwashing frequency and total number of detected chemicals; (3) self-reported gardening in the first trimester and total number of detected pesticides and concentrations of N,N-Diethyl-m-toluamide, benzyl benzoate, permethrin, di-n-butyl phthalate, and diethyl phthalate [phthalates are sometimes included as inert ingredients in pesticide formulations (35)]; (4) parity and triphenyl phosphate, as flame retardants may be present in infant products already in use in the home (36); (5) season of wear and benzophenone, an ultraviolet protectant (37); (6) season of wear and total number of pesticides. Additionally, we modeled the concentrations of chemicals detected in >60% of wristbands at 12 gestational weeks as a function of covariates and season, to assess possible seasonal differences. These multivariable linear models were performed in a similar fashion as the predictor model described above, including the same covariates and the same approach to missing data imputation and pooling.

In order to improve our understanding of exposure mixtures in the study population, we applied the self-organizing map (SOM) algorithm to the chemical concentrations measured in the silicone wristbands worn at 12 gestational weeks and for chemicals detected in >60% of samples. SOMs are a computational algorithm designed for pattern recognition and visualization of high dimensional data (38, 39), which cluster observations (here, participants) with similar multivariable profiles (here, chemical pollutant concentrations). Clusters are organized in a spatially correlated topology that aids in the visualization and interpretation of the cluster solution (38–40). SOMs have been shown to perform well when compared to traditional clustering tools (e.g., k-means), and their application to multipollutant data has been previously detailed in Pearce et al. (40). We implemented the SOM as a cluster analysis tool, as we sought to group participants with similar multipollutant exposures. As such, we guided our SOM fit using a collection of established cluster statistics in order to determine which SOM dimensions best captured the multipollutant grouping structure in our data; these statistics included the ratio of within-cluster to between-cluster of sum of squared error, the Calinski-Harabasz Index, the Dunn Index, average Silhouette length, and the Pearson Gamma statistic (41). We evaluated SOMs ranging from 4 to 25 profiles and determined final size based on the fit with most agreement across cluster statistics. Once we fit the final SOM, we assessed the distributions of the chemical concentrations in each of the profiles and examined demographic and wristband characteristics between the profiles.

In correlation analyses, multivariable linear regression models, and SOMs, we transformed the chemical concentrations to account for potential batch effects and reduce the influence of extreme observations. Specifically, we subtracted the batch-specific median value of each chemical and then standardized chemical concentrations to a mean of 0 and a standard deviation of 1.

All analyses were performed using R version 3.6 (R Core Team, Vienna, Austria) (42). The code used for this analysis may be accessed at https://github.com/BTDPhD/Frontiers-Wristbands-NHBCS.

## Results

### Study Sample

Among participants enrolled in NHBCS between March 2017 and December 2018, we obtained wristbands at ~ 12 gestational weeks from 255 women; of these, 20 wore a second wristband at ~24 gestational weeks (Table 1). For the wristband at 12 gestational weeks, more than 90% of women offered a wristband were willing to participate; among women invited to wear a second wristband later in pregnancy (~24 gestational weeks), this response rate was 84%. Participants who wore wristbands at 12 gestational weeks were generally well-educated, likely to be married, and primarily White Non-hispanic, reflective of the underlying population utilizing the study clinics. The subset of participants who wore wristbands at both time points (*n* = 20) was similar to the larger sample who wore wristbands only at 12 gestational weeks (*n* = 255) with respect to measured covariates.

**Table 1:** Characteristics of study participants who wore wristbands at either 12 or 24 gestational weeks.

			**12 Gestational weeks**	**24 Gestational weeks**
			***n* = 255**	***n* = 20**
Maternal characteristics	Age at enrollment (years)	Median (IQR)	31 (28, 35)	31 (27, 34)
	BMI (kg/m^2^)	Median (IQR)	25 (22, 30)	27 (25, 33)
	Educational attainment	Less than college graduate	45 (25)	6 (33)
		College graduate	79 (44)	8 (44)
		Any post-graduate	55 (31)	4 (22)
	Relationship status	Married	150 (82)	14 (78)
		Unmarried	33 (18)	4 (22)
	Race and ethnicity	White non-Hispanic	200 (78)	17 (85)
		Other	55 (22)	3 (15)
	Parity	0	90 (47)	10 (50)
		≥1	103 (53)	10 (50)
	Smoke exposure during pregnancy	None	153 (86)	18 (95)
		First- or second-hand exposure	24 (14)	1 (5)
Wristband characteristics	Gestational age start (weeks)	Median (IQR)	13 (12, 15)	24 (24, 26)
	Duration worn (days)	Median (IQR)	7.0 (6.9, 7.1)	7.0 (7.0, 7.3)
	Wristband size	Regular	125 (49)	16 (80)
		Small	130 (51)	4 (20)
	Season of wear	Fall	92 (37)	0 (0)
		Spring	63 (25)	4 (20)
		Summer	46 (18)	11 (55)
		Winter	50 (20)	5 (25)

*Missing values: BMI (12 gestational weeks, n = 50); educational attainment (12 gestational weeks, n = 76; 24 gestational weeks, n = 2); relationship status (12 gestational weeks, n = 72; 24 gestational weeks, n = 2); parity (12 gestational weeks, n = 62); smoke exposure during pregnancy (12 gestational weeks, n = 78; 24 gestational weeks, n = 1); gestational age start (12 gestational weeks, n = 8); season of wear (12 gestational weeks, n = 4)*.

*IQR, interquartile range*.

*Values indicate N (%) unless otherwise noted*.

### Chemicals Measured in Silicone Wristbands

For the first wristband in pregnancy, participants were provided the wristbands at a median of 13 [interquartile range (IQR): 12, 15] gestational weeks, wore the wristbands for a median of 7.0 (IQR: 6.9, 7.1) days, and approximately equal numbers of participants selected small sized wristbands (51%) and regular sized wristbands (49%). For the second wristband in pregnancy, participants were provided the wristbands at a median of 24 (IQR: 24, 26) gestational weeks, wore the wristbands for a median of 7.0 (IQR: 7.0, 7.3) days, and most participants selected regular sized wristbands (80%).

Of over 1,500 chemicals tested, 199 were detected in at least one of the wristbands worn at either 12 gestational weeks (201 detected at either 12 or 24 gestational weeks) (Supplementary Table 1). Among wristbands worn at 12 gestational weeks, 16 chemicals were detected in >60% of wristbands (Table 2); these 16 chemicals primarily included chemicals used in commerce (eight chemicals), chemicals in personal care products (eight chemicals), pesticides (five chemicals), and five phthalates. Notably, 153 chemicals were detected in <10% of wristbands and 66 chemicals were unique to a single participant.

**Table 2:** Descriptive statistics of chemical concentrations (ng/g silicone) frequently detected *in silicone* wristbands worn at 12 or 24 gestational weeks.

		**12 Gestational Weeks**		**24 Gestational Weeks**			
		**(n** **=** **255)**		**(*****n*** **=** **20)**			
**Chemical**	**CASN**	**% Detected**	**Median (IQR)**	**% Detected**	**Median (IQR)**	**ICC (95% CI) for 12 and 24 Gestational Weeks^a^**	**MyExposome Classification**
Di-n-butyl phthalate	84-74-2	99	4,580 (2,440, 8490)	100	8,790 (5,935, 14,725)	0.09 (0.00, 0.50)	Chemicals in commerce, personal care products, pesticides
Galaxolide	1222-05-5	98	6,930 (2,380, 16,000)	90	7,470 (3,028, 19,250)	0.58 (0.26, 0.75)	Chemicals in commerce, personal care products
Diisobutyl phthalate	84-69-5	97	4,810 (2,445, 8,335)	80	6,680 (768, 10,800)	0.62 (0.14, 0.78)	Chemicals in commerce
Butyl benzyl phthalate	85-68-7	94	2,650 (1,115, 8,230)	80	7,385 (3,038, 13,675)	0.59 (0.00, 0.83)	Chemicals in commerce
Lilial	80-54-6	90	1,150 (332, 2,895)	90	1,570 (673, 8978)	0.69 (0.27, 0.93)	Personal care products
Benzyl salicylate	118-58-1	89	5,050 (1,755, 12,800)	95	12,750 (4,382, 17,875)	0.13 (0.00, 0.49)	Personal care products
Tonalide	1506-02-1	83	297 (76, 1,065)	80	564 (196, 3,165)	0.68 (0.27, 0.91)	Personal care products
N,N-Diethyl-m-toluamide	134-62-3	83	715 (196, 1,710)	95	2,830 (1,027, 13,925)	0.03 (0.00, 0.47)	Pesticides
Benzophenone	119-61-9	82	220 (104, 422)	80	845 (330, 1,138)	0.22 (0.00, 0.78)	Chemicals in commerce, personal care products
Ethylene brassylate	105-95-3	82	4,580 (592, 13,600)	90	6,825 (1,595, 14,600)	0.22 (0.04, 0.79)	Personal care products
Benzyl benzoate	120-51-4	82	2,570 (763, 8,840)	85	7,860 (3,772, 17,425)	0.09 (0.00, 0.57)	Pesticides
Di-n-nonyl phthalate	84-76-4	67	662 (0, 2,415)	70	6,205 (0, 8,835)	0.13 (0.00, 0.62)	Chemicals in commerce
Permethrin	52645-53-1	67	265 (0, 1285)	70	576 (0, 871)	0.08 (0.00, 0.54)	Pesticides
Diethyl phthalate	84-66-2	64	777 (0, 2,520)	100	3,075 (2,165, 12,425)	0.14 (0.00, 0.55)	Chemicals in commerce, pesticides
Butylated hydroxyanisole	25013-16-5	64	72 (0, 174)	10	0 (0, 0)	0.00 (0.00, 0.10)	Personal care products, Pharmacological
2,4-Di-tert-butylphenol	96-76-4	61	203 (0, 972)	60	420 (0, 1,075)	0.22 (0.00, 0.77)	Chemicals in commerce
Triphenyl Phosphate	115-86-6	58	163 (0, 580)	75	504 (160, 1,580)	0.33 (0.00, 0.58)	Chemicals in commerce, flame retardants

a *Intraclass correlation coefficients and corresponding 95% CIs between chemical concentrations measured in wristbands worn at 12 and 24 gestational weeks. Chemical concentrations median standardized to batch, and centered (mean = 0) and scaled (standard deviation = 1)*.

*CASN, Chemical Abstracts Service Number; ICC, intraclass correlation coefficient; IQR, interquartile range*.

Among wristbands worn at 12 gestational weeks, a maximum of 37 chemicals was detected in a single wristband and a median of 23 (IQR: 20, 26) chemicals was detected among all wristbands (Supplementary Table 2). The most commonly detected chemical classes were chemicals in commerce (median: 12; IQR: 10, 13), chemicals in personal care products (median: 10; IQR: 8, 11), and pesticides (median: 6; IQR: 5, 8). Slightly fewer chemicals were detected in wristbands at 24 gestational weeks, though patterns were similar to those observed in the 12 gestational week wristbands (Supplementary Table 2).

**Figure 1:**
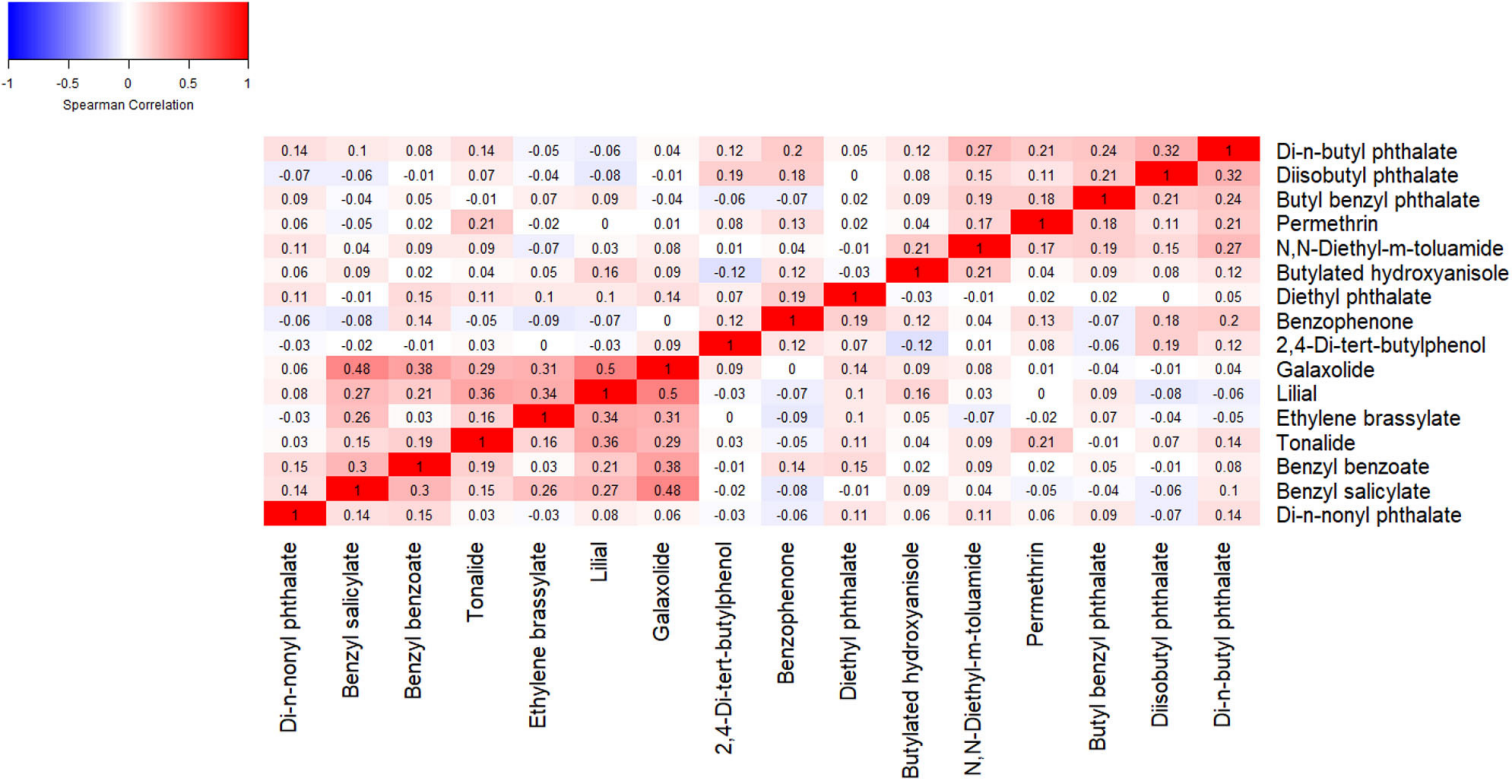
Spearman correlation coefficients among chemical concentrations detected in >60% of wristbands worn at 12 gestational weeks.

Spearman correlations among chemicals frequently detected in wristbands worn at 12 gestational weeks ranged from −0.12 to 0.50, with a median correlation of 0.08 (IQR: 0.00, 0.15) (Figure 1).

For temporal consistency between chemical concentrations measured in wristbands worn at ~12 and 24 gestational weeks, ICCs ranged from 0.00 (95% CI: 0.00, 0.10) for butylated hydroxyanisole to 0.69 (95% CI: 0.27, 0.93) for lilial (Table 2). Among the 17 frequently detected chemicals, 71% had poor reproducibility across timepoints and 29% had fair reproducibility.

### Predictors of Chemicals Measured in Silicone Wristbands

We examined whether personal characteristics or season of collection related to the number of chemicals detected in the wristband. In a multivariable linear regression model (Table 3), higher educational attainment was associated with fewer chemical exposures, albeit with limited statistical precision; relative to participants who did not graduate college, college graduates had 0.27 fewer (95% CI: −1.95, 1.42) chemicals detected in their wristbands and participants with any post-graduate education had an estimated 1.40 fewer (95% CI: −3.53, 0.73). There were differences in the number of chemicals detected by season, where wristbands particularly worn in summer (β = −2.32; 95% CI: −4.02, −0.61) and winter (β = −1.76; 95% CI: −3.32, −0.21) had fewer chemicals detected than wristbands worn in fall. Age, BMI, relationship status, race and ethnicity, parity, smoke exposure, and gestational age at start were not clearly associated with the number of chemicals detected.

**Table 3:** Multivariable linear regression model of total number of chemicals detected in wristbands worn at 12 gestational weeks.

		**Beta (95% CI)**
Age at enrollment (years)		−0.05 (−0.19, 0.09)
BMI (kg/m^2^)		0.00 (−0.08, 0.08)
Educational attainment	Less than college graduate	Ref
	College graduate	−0.27 (−1.95, 1.42)
	Any post-graduate	−1.40 (−3.53, 0.73)
Relationship status	Married	Ref
	Unmarried	0.71 (−1.13, 2.56)
Race and ethnicity	White non-hispanic	Ref
	Other	−0.05 (−1.40, 1.29)
Parity	0	Ref
	≥1	−0.38 (–**1**.72, 0.96)
Smoke exposure during pregnancy	None	Ref
	First- or second-hand exposure	0.71 (−1.20, 2.61)
Gestational age start (weeks)	Median (IQR)	−0.02 (−0.13, 0.09)
Season of wear	Fall	Ref
	Winter	−1.76 (−3.32, −0.21)
	Spring	−0.99 (−2.46, 0.49)
	Summer	−2.32 (−4.02, −0.61)

*Chemical concentrations median standardized to batch, and centered (mean = 0) and scaled (standard deviation = 1)*.

*CI, confidence interval; IQR, interquartile range*.

We further examined factors we hypothesized may be related to specific exposures (Supplementary Table 3). We observed that nail polish use in the first trimester was associated with a greater number of detected chemicals in personal care products (β = 0.43; 95% CI: −0.22, 1.09) but with wide confidence limits, and also higher concentrations of triphenyl phosphate (β = 0.27; 95% CI: 0.00, 0.55), and more weakly with phthalate concentrations. Gardening in the first trimester was weakly associated with the total number of detected pesticides (β = 0.28; 95%: −0.34, 0.91), but not with specific pesticides. Handwashing was not strongly associated with the total number of detected chemicals, and parity was not strongly related with triphenyl phosphate concentrations. The total number of detected pesticides was highest among wristbands worn in fall, and lowest among those worn in winter (β = −0.82; 95% CI: −1.51, −0.13). Relative to fall, benzophenone concentrations were highest in summer (β = 0.21; 95% CI: −0.20, 0.63) and lowest in winter (β = −0.05; 95% CI: −0.42, 0.32). Other chemical concentrations varied with respect to season (Supplementary Table 4); for example, benzyl salicylate (a fragrance and UV-blocker) was highest in spring (relative to fall; β = 0.42; 95% CI: −0.08, 0.75), ethylene brassylate (a synthetic musk) was highest in winter (relative to fall; β = 0.42; 95% CI: 0.06, 0.78), 2,4-Di-tert-butylphenol (used to produce consumer products) was highest in spring (relative to fall; β = 0.45; 95% CI: 0.12, 0.79), and diethyl phthalate (used to produce consumer products) was highest in spring (relative to fall; β = 0.35; 95% CI: 0.00, 0.69).

### Multipollutant Exposure Profiles

**Figure 2:**
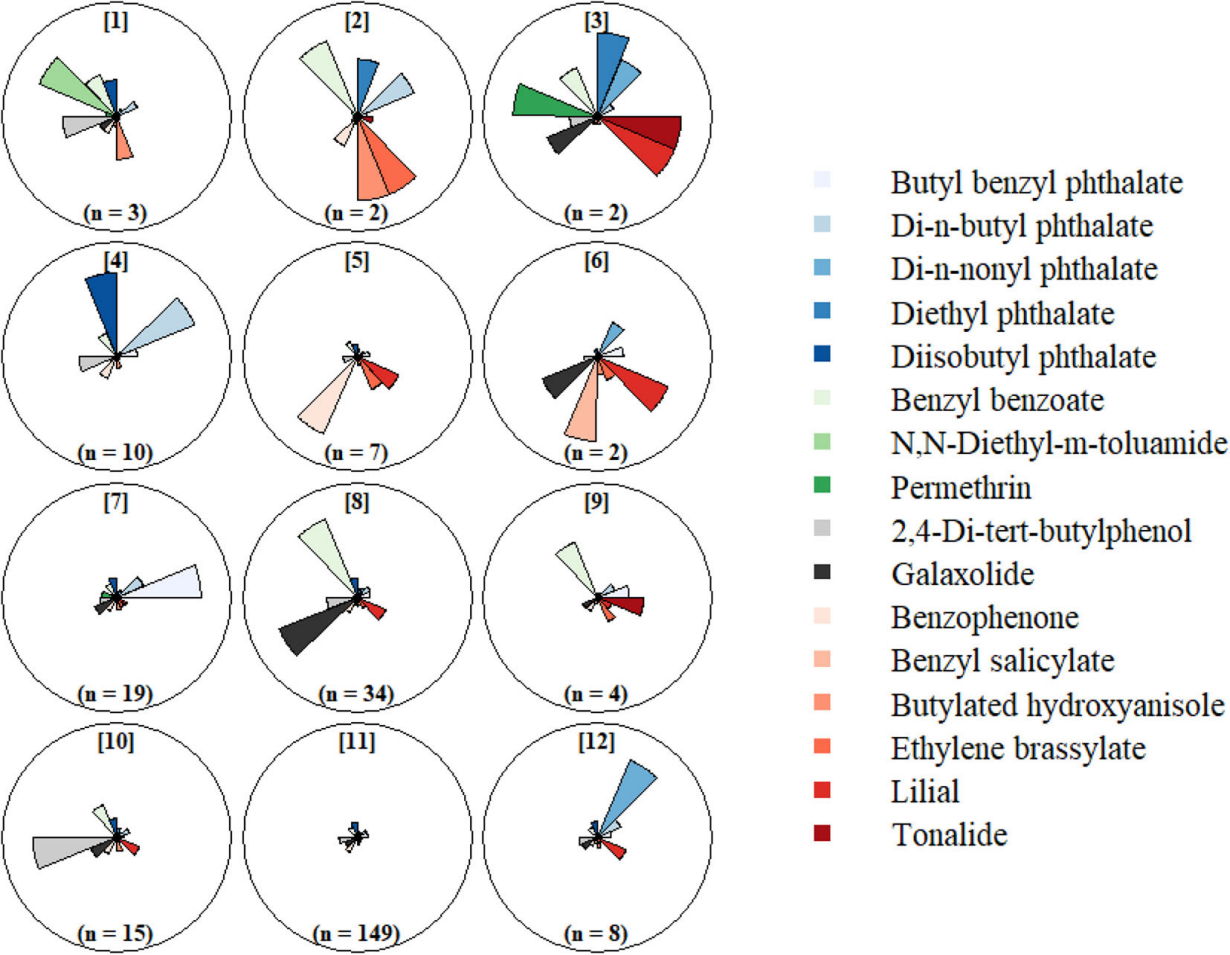
Self-organizing map (SOM) of chemical concentrations detected in >60% of silicone wristbands worn at 12 gestational weeks. Chemical concentrations standardized for batch and centered (mean = 0) and scaled (SD = 1).

Results from our clustering statistics revealed that 3 ×4 SOM consisting of 12 profiles best identified the grouping structure in the chemical concentration data. The resulting 12 profiles (Figure 2, Supplementary Table 5) revealed a broad range of multipollutant exposure patterns with highly variable frequencies. The most common exposure profile (Profile 11, 58%) included participants with relatively low exposure levels for all chemicals detected in their wristbands. Exposure scenarios exhibiting low-to-moderate concentrations for most chemicals and relatively high concentrations for one or two chemicals were captured by profiles 4, 7, 8, and 10; these scenarios were less frequent (*n* = 10, 19, 34, and 15, respectively). Specific distinctions are that Profile 4 was exceptional for high diisobutyl phthalate and high di-n-butyl phthalate concentrations, Profile 7 was exceptional for high butyl benzyl phthalate concentrations, Profile 8 was exceptional for high galaxolide and benzyl benzoate concentrations, and Profile 10 was exceptional for high 2,4-Di-tert-butylphenol concentrations. The least common (i.e., rare) scenarios (*n* < 10) were characterized by combinations of very low and very high chemical concentrations.

We further evaluated characteristics associated with SOM profiles containing ≥10 participants (Table 4). Participants in Profile 4 (high diisobutyl phthalate and di-n-butyl phthalate) were all White Non-Hispanic and more likely to be parous than participants in other profiles. Participants in Profile 7 (high butyl benzyl phthalate) had the highest proportion of non-White Non-hispanic (37%) and also had higher median BMI (26.5). Profile 8 (high galaxolide and benzyl benzoate) had a higher proportion of non-college graduates (40%) as compared to other profiles and these participants began wearing their wristbands somewhat later in pregnancy. Participants in Profile 10 (high 2,4-di-tert-butylphenol) had a lower BMI and higher educational attainment than those in other profiles. Season of wear varied substantially across profiles. No noteworthy characteristics were associated with Profile 11, the most common profile.

**Table 4:** Distributions of sociodemographic characteristics and wristband factors across SOM profiles.

		**Profile**				
		**4 (*n* = 10)**	**7 (*n* = 19)**	**8 (*n* = 34)**	**10 (*n* = 15)**	**11 (*n* = 149)**
Age at enrollment (years)	Median	32.1	30.0	30.2	30.4	31.9
BMI (kg/m^2^)	Median	24.3	26.5	24.4	23.6	24.2
Educational attainment	% < College graduate	20%	31%	40%	23%	22%
	% College graduate	40%	46%	40%	15%	46%
	% Any post-graduate	40%	23%	20%	62%	31%
Relationship status	% Married	80%	86%	67%	85%	84%
	% Single	20%	14%	33%	15%	16%
Race and ethnicity	% White non-Hispanic	100%	63%	79%	93%	76%
	% Other	0%	37%	21%	7%	24%
Parity	% 0	30%	53%	43%	60%	46%
	% ≥1	70%	47%	57%	40%	54%
Smoke exposure during pregnancy	% No	90%	93%	91%	92%	83%
	% Yes	10%	7%	9%	8%	17%
Gestational age start (weeks)	Median	12.8	12.6	13.4	13.0	12.8
Season of wear	% Winter	10%	11%	24%	27%	20%
	% Spring	60%	32%	26%	53%	17%
	% Summer	0%	26%	18%	0%	19%
	% Fall	30%	32%	32%	20%	43%
Nail polish use^a^	% Yes	30%	62%	76%	77%	44%
	% No	70%	38%	24%	23%	56%
Handwashing (times per day)^b^	Median	10.0	10.0	10.0	10.0	9.0
Gardening^c^	% Yes	20%	29%	14%	15%	30%
	% No	80%	71%	86%	85%	70%

a *Reported first trimester nail polish use (hand or foot)*.

b *Reported average number of times handwashing per day*.

c *Reported gardening in first trimester*.

## Discussion

We used silicone wristbands to investigate ambient exposures to chemical pollutants during pregnancy in a cohort of pregnant women living in rural Northern New England. The silicone wristbands functioned as passive environmental samplers, which supported the assessment of exposure to 1,530 chemicals during an ~1-week period at ~12 gestational weeks. We used data from these wristbands to learn about chemical exposures in this population, including common chemical exposures, relations between chemical exposures, predictors of chemical exposures, and common chemical exposure profiles. Our findings suggest that these silicone wristbands can be successfully deployed within sensitive populations to assess multipollutant exposures, extending the potential uses of this technology beyond previously successful investigations (15, 16, 18, 21–23).

The assay used to measure chemicals in the wristbands is capable of measuring concentrations of 1,530 pollutants. Of these 1,530 pollutants, we detected 201 unique chemicals in wristbands worn at two different points in pregnancy. Importantly, a modest subset of chemicals was detected in a majority of wristbands (i.e., 21 chemicals in >50% of wristbands worn at 12 gestational weeks), and many chemicals were detected in only a single wristband (i.e., 66 chemicals in wristbands worn at 12 gestational weeks). The chemicals detected most frequently included several chemicals in consumer products, chemicals in personal care products, pesticides, and notably five phthalates; the less frequently detected chemicals reflected a greater diversity of pollutant classes. Only a subset of the 1,530 chemicals assayed were detected, which is plausibly due to a combination of both true non-exposure and insufficient sensitivity of the analytic method for certain chemicals. Indeed, at this time there remains a meaningful tradeoff between breadth of chemicals assayed and method sensitivity, which may improve in the future. Nonetheless, we obtained quantitative measurements of exposure to over 200 chemicals in our study population with a single, non-invasive, and minimally burdensome instrument, representing in many ways a tremendous advancement in personal exposure monitoring.

We investigated the reproducibility of exposures measured in wristbands at two points in pregnancy (12 and 24 gestational weeks). Several chemicals demonstrated fair to good reproducibility at the two time points (e.g., lillial, tonalide), while many others demonstrated poor reproducibility (e.g., butylated hydoxyanisole, N,N-Diethyl-m-toluamide). Some of the chemicals with good reproducibility may be classified as chemicals in personal care products, which suggests that chemical exposures from daily behaviors may be reasonably consistent over time, and is perhaps unsurprising given that it has been estimated that women in the U.S. typically use an average of 12 personal care products each day (43). Notably, some phthalate concentrations demonstrated fair to good reproducibility (i.e., diisobutyl phthalate, butyl benzyl phthalate), whereas others were poorly reproducible (i.e., di-n-butyl phthalate, di-n-nonyl phthalate, and diethyl phthalate); as investigators seek methods to assess exposures to these ubiquitous (44, 45) and quickly-metabolized compounds (46, 47), these data suggest that the silicone wristbands may offer an alternative to capturing some, but not all, phthalates. Pesticides as a class tended to have the weakest reproducibility, which may have been expected given that the assessments of exposure were performed ~1 season apart and pesticide use likely varies by season. The chemical with the poorest reproducibility, butylated hydroxyanisole, is a food preservative (48) and concentrations of this chemical were highest in winter and fall and lowest in spring and summer, perhaps indicating a shift away from fresh foods in the colder seasons. Importantly, we had a small sample size for assessing reproducibility of exposures over the course of pregnancy, so these findings must be cautiously interpreted and serve primarily to generate hypotheses for future studies.

We sought to identify predictors of chemical exposures in our study population. First, we used multivariable linear regression to predict the total number of chemicals detected in a participant's wristband using several sociodemographic characteristics as predictors. We observed that few factors were strongly associated with total number of detected chemicals, with some evidence of an inverse association with education and variability by season. Wristbands worn in fall contained the most unique chemicals and wristbands worn in winter and summer contained significantly fewer. Seasonal trends in exposure are not uncommon among environmental exposures [e.g., air pollution (49), pesticides (50), and flame retardants (51, 52)]; however, seasonal trends are likely chemical specific and data pertaining to seasonal trends of the chemicals frequently detected in the wristbands are limited. Further assessment of seasonal trends in personal exposures to chemical mixtures may be a fruitful area for future research. Previous studies of sociodemographic predictors of chemical exposures have noted relations with factors such as age, race/ethnicity, educational attainment, marital status, and parity, though the directionality of these relations (i.e., associated with more or less exposure) varies considerably with respect to the chemical pollutant of interest and study population (24–27). A possible reason that we did not observe strong sociodemographic predictors of total number of chemicals detected is that total number of detects is a crude measure of chemical exposures and does not account for variability in the chemical constituents of a participant's exposure milieu. Additionally, our study sample is rather homogenous with respect to potential predictors of chemical exposures; e.g., our study population includes a high proportion of participants who are White Non-hispanic, college educated, and married, and relatively few participants with reported first or second hand smoke exposure.

We also investigated characteristics and behaviors in relation to specific chemical exposures selected *a priori* for their plausible relations. For example, we observed that nail polish use was associated with higher triphenyl phosphate concentrations, which is found in certain nail polishes (33). Benzophenone concentrations were highest in summer, and benzophenone is a UV-blocker found in many skincare products (37). However, other hypothesized relations between characteristics and behaviors and pollutant exposures were weak. This indicates that the wristbands may be useful for assessing some, but not all, characteristics or behaviors suspected to increase specific exposures. To note, we had hoped to investigate other *a priori* hypotheses but were unable to investigate these due to low detection frequencies of the related chemicals in the wristband samplers; e.g., total flame retardants and parity due to hypothesized presence of these chemicals in children's products (36, 53–55) and total polycyclic aromatic hydrocarbons with reported woodstove use (56–58).

Lastly, we employed self-organizing maps (SOMs) to investigate multipollutant profiles, including both their chemical makeup and demographic predictors of profile membership. The SOM is an algorithm similar to the k-means clustering algorithm but with the added benefit of a spatially-correlated topology between groups (38, 39). Among other applications, SOMs can be used to classify observations into groups with similar multipollutant exposure profiles, and have been successfully applied to air quality data (40, 59). When applied to the multipollutant data in our study, the SOM included a single profile characterized by low to moderate exposures to all chemicals that contained 58% of participants. Additionally, there were several moderate sized profiles with low to moderate exposures to all but one or two chemicals, and also several small sized profiles characterized by combinations of very high and very low exposures to several chemicals. The key findings from our SOM analyses were the marked differences in frequency of low exposure combinations (quite common) and high exposure combinations (quite rare) and the large variability in the profiles defining high magnitude exposures. We find this particularly interesting as it suggests that high exposures to combinations of frequently detected pollutants is relatively uncommon and quite individualistic in our study sample. This form of complex exposure patterning could complicate study of health effects of mixtures and needs to be examined further. We used this SOM to assess sociodemographic differences in multipollutant exposures and observed that the SOM profiles differed with respect to certain covariates, including educational attainment, BMI, race and ethnicity, and season of wear. In this manner, the SOMs provided a greater resolution to investigate multipollutant exposures than the initial multivariable linear regression model of total number of detected chemicals.

Although more than half of women in our study were placed into a single profile of low exposure to the most frequently detected chemicals, the remaining exposure profiles were more variable and included few women, and a majority of chemicals were detected in a small number wristbands. Such findings pose opportunities and challenges for multipollutant research. As an opportunity, public health initiatives to reduce harm from environmental pollutants may benefit from increased attention to the subset of highly prevalent pollutants and multipollutant mixtures, which may be prioritized for analysis with respect to health outcomes. As a challenge, the specific nature of multipollutant profiles and high inter-individual variability in exposure patterns limits our statistical power to study these highly specific mixtures in epidemiological settings. Future investigations with greater sample sizes may be better powered to study such mixtures, including demographic predictors of the mixtures and their relations with health outcomes.

A limited number of recent studies have similarly employed clustering algorithms to identify multipollutant exposure profiles in pregnant populations. Kalloo et al. (25) used k-means clustering to investigate profiles of exposure to a diverse set of 41 chemicals in a cohort of pregnant women (*n* = 389). The clustering procedure identified three groups of women, which could be described as reflecting typically high (*n* = 106), intermediate (*n* = 158), and low (*n* = 125) exposures to most chemicals. In a second study, Carroll et al. (60) employed latent class analysis to cluster pregnant women (*n* = 460) according to 15 biomarkers of exposure to phthalates and parabens. The investigators identified four groups of women, which may be described as “low exposure,” “low phthalates, “high parabens,” “high phthalates, low parabens,” and “high exposure.” Similar to our study, both works evaluated demographic predictors of profile membership; whereas Carroll et al. tended to observe higher exposure among sociodemographically disadvantaged populations, the results of Kalloo et al. suggested the converse, highlighting that patterns of multipollutant exposures may be population-specific. In addition to demographic differences in profiles, Carroll et al. additionally investigated and observed differences in oxidative stress biomarkers between the “low exposure” and “high exposure” groups, demonstrating how multipollutant profiles can be linked to health outcomes. These studies and our own illustrate the potential value of applying clustering algorithms to multipollutant exposure assessments as a means to learn about common combinations of exposures in study populations, where predictors of profile membership and linkage to outcomes may be used to identify vulnerable populations and health effects of exposure, respectively.

Our study provides support to the utility of silicone wristbands as means to assess chemical exposures in pregnancy cohorts. From a practical perspective, we observed high participation rates with the wristbands in our cohort, and study coordinators provided anecdotal reports indicating that many participants were enthusiastic to wear the wristbands and curious about the data generated by them. However, there is considerable difficulty in risk communication regarding such a broad array of chemicals, including the communication of potential toxicity, likely sources of exposure, and both absolute and relative exposure levels (61, 62). Further, reference values indicating “safe” levels of exposures are not available for the wristband data as they are for traditional biomarker measures of many, though certainly not all, chemicals. Studies may choose to focus on reporting results of particularly common chemicals, and also report chemicals unique to participants; however, this does not overcome the limited toxicological data that is available for many chemicals, which can only be overcome by additional research.

Our study has important limitations. First, while a promising means for passive and non-invasive exposure assessment, the wristbands may not capture exposures from all sources and pathways (notably, exposures from diet may be poorly captured). As such, the wristbands and multipollutant screen employed in our study may have underestimated exposure to chemicals whose exposure pathways are primarily dietary; e.g., bisphenol A is detected with high frequencies among members of the general population of the United States using biomarkers (indicating highly prevalent exposure) (63, 64), though it was detected in <3% of wristbands using the analytical method employed in this study (however, more sensitive chemical analytic methods are available for certain analytes). Further, as with other passive monitoring technologies, differences in individual variability of systemic absorption and metabolism of chemical exposures are not reflected through this method of exposure assessment (65, 66). However, relative to traditional biomarkers, the wristbands have the considerable advantage of enabling the assessment of multitudes of chemical pollutants in a single passive instrument. While previous studies have demonstrated promising relations between specific classes of exposures [e.g., PAHs (16), nicotine (67), organophosphate flame retardants (21, 68)] assessed in wristbands and traditional biomarkers of exposure, to our knowledge, no work has yet assessed relations between the broad screen of chemicals used in our study with traditional biomarkers, and this is an important area for future research.

Our study also has notable strengths. Our study included over 100 participants and used a novel non-invasive passive monitoring to identify exposure patterns using innovative methods. To our knowledge, ours is the first study to employ silicone wristbands to assess multipollutant exposures in a prospective pregnancy cohort and to evaluate repeated measures.

For future directions, it will be valuable to assess temporal consistency of chemical concentrations measured in the wristbands in larger samples, which may allow for investigation of interesting temporal trends (e.g., across pregnancy, across seasons). Additionally, further comparison of chemicals measured in wristbands to other validated exposure metrics (e.g., biomarkers, active samplers) may provide greater insight regarding which chemicals may be best measured in the wristbands. Lastly, it will be beneficial for other pregnancy cohorts to employ the wristbands, so that our study findings may be corroborated.

## Data Availability Statement

Per the NIH-approved ECHO Data Sharing Policy, ECHO-wide data have not yet been made available to the public for review/analysis. Requests to access the datasets should be directed to the ECHO Data Analysis Center, ECHO-DAC@rti.org. Requests to access the datasets should be directed to ECHO-DAC@rti.org.

## Ethics Statement

The studies involving human participants were reviewed and approved by Committee for the Protection of Human Subjects at Dartmouth College. The patients/participants provided their written informed consent to participate in this study.

## Author Contributions

MR and MK designed the study and conceived the statistical analyses, participated in interpretation of results, and assisted in drafting of the manuscript. BD refined the statistical plan, performed the statistical analyses, and drafted the manuscript under the supervision of MR. JP assisted in the analysis plan and provided technical expertise to support of the implementation and interpretation of the SOMs. KA provided feedback on the design of the exposure assessment, assisted in drafting the laboratory methods, and helped with the interpretation of results. MK was the principal investigator of the NHBCS and provided oversight to study, recruitment, specimen and wrist band collection and laboratory analyses, data management, and analysis file construction with support from KA and MR. All authors provided critical feedback and revisions to the manuscript and approved the content of the final publication.

## Conflict of Interest

KA, an author of this research, discloses a financial interest in MyExposome, Inc., which is marketing products related to the research being reported. The terms of this arrangement have been reviewed and approved by OSU in accordance with its policy on research conflicts of interest. The remaining authors declare that the research was conducted in the absence of any commercial or financial relationships that could be construed as a potential conflict of interest.

## References

[1] Woodruff TJ Zota AR Schwartz JM. Environmental chemicals in pregnant women in the United States: NHANES 2003–2004. Environ Health Perspect. (2011) 119:878–85. 10.1289/ehp.1002727 21233055 PMC3114826

[2] Louis GMB Yeung E Kannan K Maisog J Zhang C Grantz KL. Patterns and variability of endocrine-disrupting chemicals during pregnancy: implications for understanding the exposome of normal pregnancy. Epidemiology. (2019) 30:S65–S75. 10.1097/EDE.0000000000001082 31569155 PMC6777854

[3] Opinion AC Exposure to toxic environmental agents. Fertil Steril. (2013) 100:931–4. 10.1016/j.fertnstert.2013.08.043 24070500 PMC9060637

[4] Rice D Barone S Jr. Critical periods of vulnerability for the developing nervous system: evidence from humans and animal models. Environ Health Perspect. (2000) 108 (Suppl. 3):511–33. 10.1289/ehp.00108s3511 10852851 PMC1637807

[5] Selevan SG Kimmel CA Mendola P. Identifying critical windows of exposure for children's health. Environ Health Perspect. (2000) 108 (Suppl. 3):451–5. 10.1289/ehp.00108s3451 10852844 PMC1637810

[6] Haddow JE Palomaki GE Allan WC Williams JR Knight GJ Gagnon J. Maternal thyroid deficiency during pregnancy and subsequent neuropsychological development of the child. N Engl J Med. (1999) 341:549–55. 10.1056/NEJM199908193410801 10451459

[7] Gore AC Martien KM Gagnidze K Pfaff D. Implications of prenatal steroid perturbations for neurodevelopment, behavior, and autism. Endoc Rev. (2014) 35:961–91. 10.1210/er.2013-1122 25211453 PMC4234775

[8] Barr DB Bishop A Needham LL. Concentrations of xenobiotic chemicals in the maternal-fetal unit. Reprod Toxicol. (2007) 23:260–6. 10.1016/j.reprotox.2007.03.003 17386996

[9] Needham LL Grandjean P Heinzow B Jørgensen PJ Nielsen F Patterson DG Jr. Partition of environmental chemicals between maternal and fetal blood and tissues. Environ Sci Technol. (2011) 45:1121–6. 10.1021/es1019614 21166449 PMC3031182

[10] Aylward L Hays S Kirman C Marchitti S Kenneke J English C. Relationships of chemical concentrations in maternal and cord blood: a review of available data. J Toxicol Environ Health B. (2014) 17:175–203. 10.1080/10937404.2014.884956 24749481

[11] Braun JM Gennings C Hauser R Webster TF. What can epidemiological studies tell us about the impact of chemical mixtures on human health? Environ Health Perspect. (2016) 124:A6–A9. 10.1289/ehp.1510569 26720830 PMC4710611

[12] Carlin DJ Rider CV Woychik R Birnbaum LS. Unraveling the health effects of environmental mixtures: an NIEHS priority. Environ Health Perspect. (2013) 121:A6–8. 10.1289/ehp.1206182 23409283 PMC3553446

[13] Hamra GB Buckley JP. Environmental exposure mixtures: questions and methods to address them. Curr Epidemiol Rep. (2018) 5:160–5. 10.1007/s40471-018-0145-0 30643709 PMC6329601

[14] Lazarevic N Barnett AG Sly PD Knibbs LD. Statistical methodology in studies of prenatal exposure to mixtures of endocrine-disrupting chemicals: a review of existing approaches and new alternatives. Environ Health Perspect. (2019) 127:026001. 10.1289/EHP2207 30720337 PMC6752940

[15] Dixon HM Armstrong G Barton M Bergmann AJ Bondy M Halbleib ML. Discovery of common chemical exposures across three continents using silicone wristbands. R Soc Open Sci. (2019) 6:181836. 10.1098/rsos.181836 30891293 PMC6408398

[16] Dixon HM Scott RP Holmes D Calero L Kincl LD Waters KM. Silicone wristbands compared with traditional polycyclic aromatic hydrocarbon exposure assessment methods. Anal Bioanal Chem. (2018) 410:3059–71. 10.1007/s00216-018-0992-z 29607448 PMC5910488

[17] O'Connell SG Kincl LD Anderson KA Silicone wristbands as personal passive samplers. Environ Sci Technol. (2014) 48:3327–35. 10.1021/es405022f 24548134 PMC3962070

[18] Kile ML Scott RP O'Connell SG Lipscomb S MacDonald M McClelland M. Using silicone wristbands to evaluate preschool children's exposure to flame retardants. Environ Res. (2016) 147:365–72. 10.1016/j.envres.2016.02.034 26945619 PMC4821754

[19] Anderson KA Points GL 3rd Donald CE Dixon HM Scott RP Wilson G. Preparation and performance features of wristband samplers and considerations for chemical exposure assessment. J Exposure Sci Environ Epidemiol. (2017) 27:551–9. 10.1038/jes.2017.9 28745305 PMC5658681

[20] Bergmann AJ Points GL Scott RP Wilson G Anderson KA. Development of quantitative screen for 1550 chemicals with GC-MS. Anal Bioanal Chem. (2018) 410:3101–10. 10.1007/s00216-018-0997-7 29552732 PMC5910463

[21] Gibson EA Stapleton HM Calero L Holmes D Burke K Martinez R. Differential exposure to organophosphate flame retardants in mother-child pairs. Chemosphere. (2019) 219:567–73. 10.1016/j.chemosphere.2018.12.008 30553217 PMC6460923

[22] Donald CE Scott RP Blaustein KL Halbleib ML Sarr M Jepson PC. Silicone wristbands detect individuals' pesticide exposures in West Africa. R Soc Open Sci. (2016) 3:160433. 10.1098/rsos.160433 27853621 PMC5108971

[23] Aerts R Joly L Szternfeld P Tsilikas K De Cremer K Castelain P. Silicone wristband passive samplers yield highly individualized pesticide residue exposure profiles. Environ Sci Technol. (2018) 52:298–307. 10.1021/acs.est.7b05039 29185731

[24] Bulka CM Bommarito PA Fry RC. Predictors of toxic metal exposures among US women of reproductive age. J Exposure Sci Environ Epidemiol. (2019) 29:597–612. 10.1038/s41370-019-0152-3 31235790 PMC6709576

[25] Kalloo G Wellenius GA McCandless L Calafat AM Sjodin A Karagas M. Profiles and predictors of environmental chemical mixture exposure among pregnant women: the health outcomes and measures of the environment study. Environ Sci Technol. (2018) 52:10104–13. 10.1021/acs.est.8b02946 30088764 PMC10105973

[26] Lewin A Arbuckle TE Fisher M Liang CL Marro L Davis K. Univariate predictors of maternal concentrations of environmental chemicals: the MIREC study. Int J Hygiene Environ Health. (2017) 220:77–85. 10.1016/j.ijheh.2017.01.001 28109710

[27] Vrijheid M Martinez D Aguilera I Ballester F Basterrechea M Esplugues A. Socioeconomic status and exposure to multiple environmental pollutants during pregnancy: evidence for environmental inequity? J Epidemiol Commun Health. (2012) 66:106–13. 10.1136/jech.2010.117408 20974841

[28] Rosner B. Fundamentals of Biostatistics. Boston, MA: Nelson Education (2015).

[29] Buuren Sv Groothuis-Oudshoorn K mice: multivariate imputation by chained equations in R. J Statistical Softw. (2010) 2010:1–68. 10.18637/jss.v045.i03

[30] Guo Y Kannan K. A survey of phthalates and parabens in personal care products from the United States and its implications for human exposure. Environ Sci Technol. (2013) 47:14442–9. 10.1021/es4042034 24261694

[31] Koniecki D Wang R Moody RP Zhu J. Phthalates in cosmetic and personal care products: concentrations and possible dermal exposure. Environ Res. (2011) 111:329–36. 10.1016/j.envres.2011.01.013 21315328

[32] Parlett LE Calafat AM Swan SH. Women's exposure to phthalates in relation to use of personal care products. J Exposure Sci Environ Epidemiol. (2013) 23:197–206. 10.1038/jes.2012.105 23168567 PMC4097177

[33] Mendelsohn E Hagopian A Hoffman K Butt CM Lorenzo A Congleton J. Nail polish as a source of exposure to triphenyl phosphate. Environ Int. (2016) 86:45–51. 10.1016/j.envint.2015.10.005 26485058 PMC4662901

[34] Young AS Allen JG Kim U-J Seller S Webster TF Kannan K. Phthalate and organophosphate plasticizers in nail polish: evaluation of labels and ingredients. Environ Sci Technol. (2018) 52:12841–50. 10.1021/acs.est.8b04495 30302996 PMC6222550

[35] US Environmental Protection Agency Pesticide Registration - Inert Ingredients Overview and Guidance. Available online from: https://www.epa.gov/pesticide-registration/inert-ingredients-overview-and-guidance

[36] Hoffman K Butt CM Chen A Limkakeng AT Jr Stapleton HM. High exposure to organophosphate flame retardants in infants: associations with baby products. Environ Sci Technol. (2015) 49:14554–9. 10.1021/acs.est.5b03577 26551726

[37] Romano ME Kalloo G Etzel T Braun JM. Seasonal variation in exposure to endocrine disrupting chemicals. Epidemiology. (2017) 28:e42. 10.1097/EDE.0000000000000696 28570386 PMC5539945

[38] Kohonen T Self-Organizing Maps. 3rd Ed. Berlin: Springer (2001) 105–11.

[39] Kohonen T. Essentials of the self-organizing map. Neural Netw. (2013) 37:52–65. 10.1016/j.neunet.2012.09.018 23067803

[40] Pearce JL Waller LA Chang HH Klein M Mulholland JA Sarnat JA. Using self-organizing maps to develop ambient air quality classifications: a time series example. Environ Health. (2014) 13:56. 10.1186/1476-069X-13-56 24990361 PMC4098670

[41] Everitt B Landau S Leese M Stahl D Cluster Analysis. 5th Ed. West Sussex, UK: Wiley (2011) 260–269. 10.1002/9780470977811

[42] R Core Team R: A language and environment for statistical computing. Vienna, Austria: R Foundation for Statistical Computing (2019).

[43] Environmental Working Group Exposures Add up - Survey Results. (2004). Available Online from: https://web.archive.org/web/20150727050213/; https:/www.ewg.org/skindeep/2004/06/15/exposures-add-up-survey-results/

[44] Wang Y Zhu H Kannan K. A review of biomonitoring of phthalate exposures. Toxics. (2019) 7:21. 10.3390/toxics7020021 30959800 PMC6630674

[45] Katsikantami I Sifakis S Tzatzarakis MN Vakonaki E Kalantzi OI Tsatsakis AM. A global assessment of phthalates burden and related links to health effects. Environ Int. (2016) 97:212–36. 10.1016/j.envint.2016.09.013 27669632

[46] Calafat AM Needham LL. Factors affecting the evaluation of biomonitoring data for human exposure assessment. Int J Androl. (2008) 31:139–43. 10.1111/j.1365-2605.2007.00826.x 17971164

[47] Johns LE Cooper GS Galizia A Meeker JD. Exposure assessment issues in epidemiology studies of phthalates. Environ Int. (2015) 85:27–39. 10.1016/j.envint.2015.08.005 26313703 PMC4648682

[48] Verhagen H Schilderman PA Kleinjans JC. Butylated hydroxyanisole in perspective. Chem Biol Interact. (1991) 80:109–34. 10.1016/0009-2797(91)90019-4 1934145

[49] Szpiro AA Sampson PD Sheppard L Lumley T Adar SD Kaufman JD. Predicting intra-urban variation in air pollution concentrations with complex spatio-temporal dependencies. Environmetrics. (2010) 21:606–31. 10.1002/env.1014 24860253 PMC4029437

[50] Smith MN Workman T McDonald KM Vredevoogd MA Vigoren EM Griffith WC. Seasonal and occupational trends of five organophosphate pesticides in house dust. J Exposure Sci Environ Epidemiol. (2017) 27:372–8. 10.1038/jes.2016.45 27553992

[51] Hoffman K Butt CM Webster TF Preston EV Hammel SC Makey C. Temporal trends in exposure to organophosphate flame retardants in the United States. Environ Sci Technol Lett. (2017) 4:112–8. 10.1021/acs.estlett.6b00475 28317001 PMC5352975

[52] Cao Z Xu F Covaci A Wu M Yu G Wang B. Differences in the seasonal variation of brominated and phosphorus flame retardants in office dust. Environ Int. (2014) 65:100–6. 10.1016/j.envint.2013.12.011 24480750

[53] Peng B Yu Z-M Wu C-C Liu L-Y Zeng L Zeng EY. Polybrominated diphenyl ethers and organophosphate esters flame retardants in play mats from China and the exposure risks for children. Environ Int. (2020) 135:105348. 10.1016/j.envint.2019.105348 31884131

[54] Ionas AC Dirtu AC Anthonissen T Neels H Covaci A. Downsides of the recycling process: harmful organic chemicals in children's toys. Environ Int. (2014) 65:54–62. 10.1016/j.envint.2013.12.019 24468634

[55] Stapleton HM Klosterhaus S Keller A Ferguson PL van Bergen S Cooper E. Identification of flame retardants in polyurethane foam collected from baby products. Environ Sci Technol. (2011) 45:5323–31. 10.1021/es2007462 21591615 PMC3113369

[56] Vicente E Vicente A Evtyugina M Oduber F Amato F Querol X. Impact of wood combustion on indoor air quality. Sci Total Environ. (2020) 705:135769. 10.1016/j.scitotenv.2019.135769 31818582

[57] de Gennaro G Dambruoso PR Di Gilio A Di Palma V Marzocca A Tutino M Discontinuous and continuous indoor air quality monitoring in homes with fireplaces or wood stoves as heating system. Int J Environ Res Public Health. (2015) 13:78 10.3390/ijerph13010078 26712773 PMC4730469

[58] Gullett BK Touati A Hays MD. PCDD/F, PCB, HxCBz, PAH, and PM emission factors for fireplace and woodstove combustion in the San Francisco Bay region. Environ Sci Technol. (2003) 37:1758–65. 10.1021/es026373c 12775046

[59] Pearce JL Waller LA Mulholland JA Sarnat SE Strickland MJ Chang HH. Exploring associations between multipollutant day types and asthma morbidity: epidemiologic applications of self-organizing map ambient air quality classifications. Environ Health. (2015) 14:55. 10.1186/s12940-015-0041-8 26099363 PMC4477305

[60] Carroll R White AJ Keil AP Meeker JD McElrath TF Zhao S. Latent classes for chemical mixtures analyses in epidemiology: an example using phthalate and phenol exposure biomarkers in pregnant women. J Exposure Sci Environ Epidemiol. (2019) 30:149–59. 10.1038/s41370-019-0181-y 31636370 PMC6917962

[61] Ramirez-Andreotta MD Brody JG Lothrop N Loh M Beamer PI Brown P. Reporting back environmental exposure data and free choice learning. Environ Health. (2016) 15:2. 10.1186/s12940-015-0080-1 26748908 PMC4707004

[62] Tomsho KS Schollaert C Aguilar T Bongiovanni R Alvarez M Scammell MK. A Mixed methods evaluation of sharing air pollution results with study participants via report-back communication. Int J Environ Res Public Health. (2019) 16:4183. 10.3390/ijerph16214183 31671859 PMC6862165

[63] Lehmler HJ Liu B Gadogbe M Bao W. Exposure to Bisphenol A, Bisphenol F, and Bisphenol S in U.S. Adults and children: the national health and nutrition examination survey 2013-2014. ACS Omega. (2018) 3:6523–32. 10.1021/acsomega.8b00824 29978145 PMC6028148

[64] Ye X Wong LY Kramer J Zhou X Jia T Calafat AM. Urinary concentrations of bisphenol A and three other bisphenols in convenience samples of U.S. Adults during 2000-2014. Environ Sci Technol. (2015) 49:11834–9. 10.1021/acs.est.5b02135 26360019 PMC7948051

[65] Klaassen CD (editor). Casarett and Doull's toxicology: the Basic Science of Poisons. 9th edn. New York, NY: McGraw-Hill (2019)

[66] Bois FY Jamei M Clewell HJ. PBPK modelling of inter-individual variability in the pharmacokinetics of environmental chemicals. Toxicology. (2010) 278:256–67. 10.1016/j.tox.2010.06.007 20600548

[67] Quintana PJ Hoh E Dodder NG Matt GE Zakarian JM Anderson KA. Nicotine levels *in silicone* wristband samplers worn by children exposed to secondhand smoke and electronic cigarette vapor are highly correlated with child's urinary cotinine. J Exposure Sci Environ Epidemiol. (2019) 29:733–41. 10.1038/s41370-019-0116-7 30728487

[68] Hammel SC Hoffman K Webster TF Anderson KA Stapleton HM Measuring personal exposure to organophosphate flame retardants using silicone wristbands and hand wipes. Environ Sci Technol. (2016) 50:4483–91. 10.1021/acs.est.6b00030 26975559 PMC4872512

