# Supplementary material for: Assessment of Multipollutant Exposures During Pregnancy Using Silicone Wristbands
Source: Front Public Health. 2020 Sep 29;8:547239. doi: 10.3389/fpubh.2020.547239 (PMC7550746; doi:10.3389/fpubh.2020.547239)
Supplement: Supplementary file 1 [file Table_1.DOCX]

Supplementary Material:

Assessment of Multipollutant Exposures during Pregnancy using Silicone Wristbands

Brett T Doherty^1*^, John L Pearce^2^, Kim A Anderson^3^, Margaret R Karagas^1^, Megan E Romano^1^ on behalf of program collaborators for Environmental Influences on Child Health Outcomes

^1^Department of Epidemiology, Geisel School of Medicine at Dartmouth, Hanover, New Hampshire, USA

^2^Department of Public Health Sciences, Medical University of South Carolina, Charleston, South Carolina, USA

^3^Department of Environmental and Molecular Toxicology, Oregon State University, Corvallis, Oregon, USA

**Contents**

Supplemental Table 1. Descriptive statistics of chemical concentrations (ng/g silicone) measured in silicone wristbands worn at 12 or 24 gestational weeks.........................................................................2

Supplemental Table 2. Summary of chemicals detected in silicone wristbands by MyExposome classification...........................................................................................................................................9

Supplemental Table 3. Multivariable linear regression of specific characteristics in relation to wristband chemical detection or chemical concentrations...................................................................10

Supplemental Table 4. Multivariable linear regression of season in relation to chemical concentrations measured in wristbands worn at 12 gestational weeks.................................................11

Supplemental Table 5. Median concentrations of chemicals measured in wristbands worn at 12 gestational weeks by SOM profile........................................................................................................12

| Supplemental Table 1. Descriptive statistics of chemical concentrations (ng/g silicone) measured in silicone wristbands worn at 12 or 24 gestational weeks. | | | | | | | | | | | | |
| --- | --- | --- | --- | --- | --- | --- | --- | --- | --- | --- | --- | --- |
|  |  | 12 Gestational Weeks  (n = 225) | | 24 Gestational Weeks  (n = 20) | | MyExposome Classification | | | | | | |
| Chemical | CASN | % Detected | Median (IQR) | % Detected | Median (IQR) | Chemicals in Commerce | Personal Care Products | Pesticides | Consumer Products | Flame Retardants | Pharmacologic | PAHs |
| Di-n-butyl phthalate | 84-74-2 | 99 | 4580 (2440, 8490) | 100 | 8790 (5935, 14725) | X | X | X |  |  |  |  |
| Galaxolide | 1222-05-5 | 98 | 6930 (2380, 16000) | 90 | 7470 (3028, 19250) | X | X |  |  |  |  |  |
| Diisobutyl phthalate | 84-69-5 | 97 | 4810 (2445, 8335) | 80 | 6680 (768, 10800) | X |  |  |  |  |  |  |
| Butyl benzyl phthalate | 85-68-7 | 94 | 2650 (1115, 8230) | 80 | 7385 (3038, 13675) | X |  |  |  |  |  |  |
| Lilial | 80-54-6 | 90 | 1150 (332, 2895) | 90 | 1570 (673, 8978) |  | X |  |  |  |  |  |
| Benzyl salicylate | 118-58-1 | 89 | 5050 (1755, 12800) | 95 | 12750 (4382, 17875) |  | X |  |  |  |  |  |
| Tonalide | 1506-02-1 | 83 | 297 (76, 1065) | 80 | 564 (196, 3165) |  | X |  |  |  |  |  |
| N,N-Diethyl-m-toluamide | 134-62-3 | 83 | 715 (196, 1710) | 95 | 2830 (1027, 13925) |  |  | X |  |  |  |  |
| Benzophenone | 119-61-9 | 82 | 220 (104, 422) | 80 | 845 (330, 1138) | X | X |  |  |  |  |  |
| Ethylene brassylate | 105-95-3 | 82 | 4580 (592, 13600) | 90 | 6825 (1595, 14600) |  | X |  |  |  |  |  |
| Benzyl benzoate | 120-51-4 | 82 | 2570 (763, 8840) | 85 | 7860 (3772, 17425) |  |  | X |  |  |  |  |
| Di-n-nonyl phthalate | 84-76-4 | 67 | 662 (0, 2415) | 70 | 6205 (0, 8835) | X |  |  |  |  |  |  |
| Permethrin | 52645-53-1 | 67 | 265 (0, 1285) | 70 | 576 (0, 871) |  |  | X |  |  |  |  |
| Diethyl phthalate | 84-66-2 | 64 | 777 (0, 2520) | 100 | 3075 (2165, 12425) | X |  | X |  |  |  |  |
| Butylated hydroxyanisole | 25013-16-5 | 64 | 72 (0, 174) | 10 | 0 (0, 0) |  | X |  |  |  | X |  |
| 2,4-Di-tert-butylphenol | 96-76-4 | 61 | 203 (0, 972) | 60 | 420 (0, 1075) | X |  |  |  |  |  |  |
| Bis(2-ethylhexyl)phthalate | 117-81-7 | 60 | 1580 (0, 56750) | 55 | 2215 (0, 95575) | X |  | X |  |  |  |  |
| Triphenyl Phosphate | 115-86-6 | 58 | 163 (0, 580) | 75 | 504 (160, 1580) | X |  |  |  | X |  |  |
| Butylated hydroxytoluene | 128-37-0 | 57 | 60 (0, 374) | 55 | 336 (0, 934) | X |  |  | X |  |  |  |
| Amyl cinnamal | 122-40-7 | 55 | 141 (0, 675) | 55 | 658 (0, 2972) |  | X |  |  |  |  |  |
| TCPP | 26248-87-3 | 52 | 63 (0, 582) | 30 | 0 (0, 862) | X |  |  |  | X |  |  |
| B-Ionone | 79-77-6 | 40 | 0 (0, 161) | 40 | 0 (0, 442) |  | X |  |  |  |  |  |
| Permethrin II | 999046-03-6 | 39 | 0 (0, 656) | 30 | 0 (0, 755) |  |  | X |  |  |  |  |
| Benzothiazole | 95-16-9 | 39 | 0 (0, 69) | 15 | 0 (0, 0) | X |  |  |  |  |  |  |
| Caffeine | 58-08-2 | 32 | 0 (0, 339) | 50 | 184 (0, 1592) |  |  |  | X |  | X |  |
| 2,4-Bis(alpha,alpha-dimethylbenzyl)phenol | 2772-45-4 | 29 | 0 (0, 48) | 0 | 0 (0, 0) | X |  |  |  |  |  |  |
| Anthracene | 120-12-7 | 27 | 0 (0, 11) | 10 | 0 (0, 0) |  |  |  |  |  |  | X |
| Coumarin | 91-64-5 | 24 | 0 (0, 0) | 0 | 0 (0, 0) |  | X |  | X |  |  |  |
| B-citronellol | 106-22-9 | 24 | 0 (0, 0) | 5 | 0 (0, 0) |  | X |  | X |  |  |  |
| Ethofenprox | 80844-07-1 | 21 | 0 (0, 0) | 0 | 0 (0, 0) |  |  | X |  |  |  |  |
| Dicyclohexyl phthalate | 84-61-7 | 20 | 0 (0, 0) | 25 | 0 (0, 215) | X |  |  |  |  |  |  |
| Citral A | 5392-40-5 | 20 | 0 (0, 0) | 0 | 0 (0, 0) |  | X |  | X |  |  |  |
| 1-Methylnaphthalene | 90-12-0 | 20 | 0 (0, 0) | 0 | 0 (0, 0) | X |  |  |  |  |  | X |
| Linalool | 78-70-6 | 18 | 0 (0, 0) | 15 | 0 (0, 0) |  | X | X |  |  |  |  |
| Di-n-octyl phthalate | 117-84-0 | 15 | 0 (0, 0) | 10 | 0 (0, 0) | X |  |  |  |  |  |  |
| Naphthalene | 91-20-3 | 15 | 0 (0, 0) | 0 | 0 (0, 0) |  |  |  |  |  |  | X |
| Exaltolide [15-Pentadecanolide] | 106-02-5 | 15 | 0 (0, 0) | 30 | 0 (0, 3540) |  | X |  |  |  |  |  |
| Cinnamal | 104-55-2 | 14 | 0 (0, 0) | 0 | 0 (0, 0) | X | X | X | X |  |  |  |
| A-Ionone | 127-41-3 | 14 | 0 (0, 0) | 15 | 0 (0, 0) |  | X |  |  |  |  |  |
| Tributyl phosphate | 126-73-8 | 13 | 0 (0, 0) | 10 | 0 (0, 0) | X |  |  |  | X |  |  |
| Tris(2-ethylhexyl) phosphate | 78-42-2 | 12 | 0 (0, 0) | 10 | 0 (0, 0) | X |  |  |  | X |  |  |
| Piperonyl butoxide | 51-03-6 | 12 | 0 (0, 0) | 10 | 0 (0, 0) |  |  | X |  |  |  |  |
| 4-Chloro-3,5-dimethylphenol | 88-04-0 | 11 | 0 (0, 0) | 10 | 0 (0, 0) | X |  |  |  |  |  |  |
| Pyrene | 129-00-0 | 11 | 0 (0, 0) | 0 | 0 (0, 0) | X |  |  |  |  |  | X |
| Quinoline | 91-22-5 | 11 | 0 (0, 0) | 0 | 0 (0, 0) | X |  |  |  |  |  |  |
| Di-n-hexyl phthalate | 84-75-3 | 10 | 0 (0, 0) | 15 | 0 (0, 0) | X |  |  |  |  |  |  |
| Dimethyl phthalate | 131-11-3 | 9 | 0 (0, 0) | 5 | 0 (0, 0) | X |  | X |  |  |  |  |
| 4-Tert-butylphenol | 98-54-4 | 9 | 0 (0, 0) | 5 | 0 (0, 0) | X |  |  | X |  |  |  |
| Triclosan | 3380-34-5 | 8 | 0 (0, 0) | 0 | 0 (0, 0) | X | X |  |  |  | X |  |
| Phenanthrene | 85-01-8 | 7 | 0 (0, 0) | 5 | 0 (0, 0) |  |  |  |  |  |  | X |
| Drometrizole | 2440-22-4 | 7 | 0 (0, 0) | 0 | 0 (0, 0) | X |  |  | X |  |  |  |
| Methoprene II | 999045-03-3 | 6 | 0 (0, 0) | 0 | 0 (0, 0) |  |  | X |  |  |  |  |
| Lyral | 31906-04-4 | 6 | 0 (0, 0) | 5 | 0 (0, 0) |  | X |  |  |  |  |  |
| Eugenol | 97-53-0 | 6 | 0 (0, 0) | 10 | 0 (0, 0) | X | X | X | X |  | X |  |
| D-Limonene | 5989-27-5 | 6 | 0 (0, 0) | 0 | 0 (0, 0) |  | X |  | X |  |  |  |
| Benzyl alcohol | 100-51-6 | 6 | 0 (0, 0) | 0 | 0 (0, 0) | X | X |  | X |  | X |  |
| 2-Methylphenanthrene | 2531-84-2 | 6 | 0 (0, 0) | 0 | 0 (0, 0) |  |  |  |  |  |  | X |
| Phantolide | 15323-35-0 | 6 | 0 (0, 0) | 0 | 0 (0, 0) |  | X |  |  |  |  |  |
| Benzyl cinnamate | 103-41-3 | 5 | 0 (0, 0) | 5 | 0 (0, 0) |  | X |  | X |  |  |  |
| 1-Methylphenanthrene | 832-69-9 | 5 | 0 (0, 0) | 0 | 0 (0, 0) |  |  |  |  |  |  | X |
| Promecarb | 2631-37-0 | 5 | 0 (0, 0) | 5 | 0 (0, 0) |  |  | X |  |  |  |  |
| Hydroxy-citronellal | 107-75-5 | 5 | 0 (0, 0) | 5 | 0 (0, 0) |  | X |  |  |  |  |  |
| Fluorene | 86-73-7 | 5 | 0 (0, 0) | 0 | 0 (0, 0) |  |  |  |  |  |  | X |
| Fipronil | 120068-37-3 | 4 | 0 (0, 0) | 0 | 0 (0, 0) |  |  | X |  |  | X |  |
| Fenobucarb | 3766-81-2 | 4 | 0 (0, 0) | 5 | 0 (0, 0) |  |  | X |  |  |  |  |
| Thymol | 89-83-8 | 4 | 0 (0, 0) | 5 | 0 (0, 0) |  |  |  |  |  | X |  |
| Acenaphthylene | 208-96-8 | 4 | 0 (0, 0) | 10 | 0 (0, 0) |  |  |  |  |  |  | X |
| Triethyl phosphate | 78-40-0 | 4 | 0 (0, 0) | 5 | 0 (0, 0) | X |  | X |  | X |  |  |
| Phthalimide | 85-41-6 | 4 | 0 (0, 0) | 10 | 0 (0, 0) | X |  | X |  |  |  |  |
| Cashmeran | 33704-61-9 | 4 | 0 (0, 0) | 10 | 0 (0, 0) |  | X |  |  |  |  |  |
| Indole | 120-72-9 | 3 | 0 (0, 0) | 5 | 0 (0, 0) | X | X |  |  |  |  |  |
| 3-Tert-butylphenol | 585-34-2 | 3 | 0 (0, 0) | 5 | 0 (0, 0) | X |  |  |  |  |  |  |
| Tri-p-tolyl phosphate | 78-32-0 | 3 | 0 (0, 0) | 0 | 0 (0, 0) | X |  |  |  |  |  |  |
| PBDE 47 | 5436-43-1 | 3 | 0 (0, 0) | 0 | 0 (0, 0) |  |  |  |  | X |  |  |
| Pyriproxyfen | 95737-68-1 | 3 | 0 (0, 0) | 10 | 0 (0, 0) |  |  | X |  |  |  |  |
| Bisphenol A | 80-05-7 | 3 | 0 (0, 0) | 0 | 0 (0, 0) | X |  |  |  |  |  |  |
| Tricresylphosphate, meta- | 563-04-2 | 2 | 0 (0, 0) | 0 | 0 (0, 0) | X |  |  |  | X |  |  |
| 1-Hydroxynaphthalene | 90-15-3 | 2 | 0 (0, 0) | 0 | 0 (0, 0) | X |  |  |  |  |  | X |
| PBDE 49 | 243982-82-3 | 2 | 0 (0, 0) | 0 | 0 (0, 0) |  |  |  |  | X |  |  |
| O-Phenylphenol | 90-43-7 | 2 | 0 (0, 0) | 0 | 0 (0, 0) | X |  | X |  |  |  |  |
| 4-Chlorophenyl isocyanate | 104-12-1 | 2 | 0 (0, 0) | 0 | 0 (0, 0) | X |  | X |  |  |  |  |
| Acenaphthene | 83-32-9 | 2 | 0 (0, 0) | 0 | 0 (0, 0) |  |  |  |  |  |  | X |
| Promecarb artifact [5-isopropyl-3-methylphenol] | 3228-03-3 | 2 | 0 (0, 0) | 0 | 0 (0, 0) |  |  | X |  |  |  |  |
| 2,6-Dimethylnaphthalene | 581-42-0 | 2 | 0 (0, 0) | 0 | 0 (0, 0) |  |  |  |  |  |  | X |
| Isobornyl thiocyanoacetate | 115-31-1 | 2 | 0 (0, 0) | 10 | 0 (0, 0) |  |  | X |  |  |  |  |
| Celestolide | 13171-00-1 | 2 | 0 (0, 0) | 5 | 0 (0, 0) |  | X |  | X |  |  |  |
| Cinerin I | 25402-06-6 | 2 | 0 (0, 0) | 0 | 0 (0, 0) |  |  | X |  |  |  |  |
| Cypermethrin-2 | 52315-07-8 | 2 | 0 (0, 0) | 0 | 0 (0, 0) |  |  | X |  |  |  |  |
| Farnesol I | 4602-84-0 | 2 | 0 (0, 0) | 0 | 0 (0, 0) |  | X |  | X |  |  |  |
| Geraniol | 106-24-1 | 2 | 0 (0, 0) | 5 | 0 (0, 0) |  | X |  | X |  |  |  |
| 3-Chloroaniline | 108-42-9 | 2 | 0 (0, 0) | 0 | 0 (0, 0) | X |  |  |  |  |  |  |
| 2-Methylnaphthalene | 91-57-6 | 2 | 0 (0, 0) | 0 | 0 (0, 0) | X |  |  |  |  |  | X |
| Diuron Metabolite [3,4-Dichlorophenyl isocyanate] | 102-36-3 | 2 | 0 (0, 0) | 0 | 0 (0, 0) | X |  |  |  |  |  |  |
| Benzo(a)pyrene-7,8-dione | 65199-11-3 | 1 | 0 (0, 0) | 0 | 0 (0, 0) |  |  |  |  |  |  | X |
| Isoeugenol | 97-54-1 | 1 | 0 (0, 0) | 0 | 0 (0, 0) |  | X |  |  |  |  |  |
| 2,6-Di-tert-butyl-4-ethylphenol | 4130-42-1 | 1 | 0 (0, 0) | 0 | 0 (0, 0) | X |  |  |  |  |  |  |
| 2,6-Di-tert-butylphenol | 128-39-2 | 1 | 0 (0, 0) | 0 | 0 (0, 0) | X |  |  |  |  |  |  |
| Methoxychlor | 72-43-5 | 1 | 0 (0, 0) | 10 | 0 (0, 0) |  |  | X |  |  | X |  |
| Hexazinone | 51235-04-2 | 1 | 0 (0, 0) | 0 | 0 (0, 0) |  |  | X |  |  |  |  |
| 2,4-Dichlorophenol | 120-83-2 | 1 | 0 (0, 0) | 0 | 0 (0, 0) | X |  | X |  |  |  |  |
| PBDE 99 | 60348-60-9 | 1 | 0 (0, 0) | 0 | 0 (0, 0) |  |  |  |  | X |  |  |
| PCB 94 | 73575-55-0 | 1 | 0 (0, 0) | 0 | 0 (0, 0) |  |  |  |  |  |  |  |
| Pentachloroanisole | 1825-21-4 | 1 | 0 (0, 0) | 0 | 0 (0, 0) |  |  | X |  |  |  |  |
| Carvone | 99-49-0 | 1 | 0 (0, 0) | 0 | 0 (0, 0) |  | X | X | X |  |  |  |
| Musk Ketone | 81-14-1 | 1 | 0 (0, 0) | 0 | 0 (0, 0) |  | X |  |  |  |  |  |
| Tolyltriazole [1H-Benzotriazole, 4-methyl-] | 29878-31-7 | 1 | 0 (0, 0) | 0 | 0 (0, 0) | X |  |  |  |  |  |  |
| 2,6-Diethylnaphthalene | 59919-41-4 | 1 | 0 (0, 0) | 0 | 0 (0, 0) |  |  |  |  |  |  | X |
| Benzo[a]fluorene | 238-84-6 | 1 | 0 (0, 0) | 0 | 0 (0, 0) |  |  |  |  |  |  | X |
| Benzo[b]fluorene | 243-17-4 | 1 | 0 (0, 0) | 0 | 0 (0, 0) |  |  |  |  |  |  | X |
| 2,6-Dimethylphenol | 576-26-1 | 1 | 0 (0, 0) | 0 | 0 (0, 0) | X |  |  | X |  |  |  |
| 2-Chlorosyringaldehyde | 76341-69-0 | 1 | 0 (0, 0) | 10 | 0 (0, 0) | X |  |  |  |  |  |  |
| Methyleugenol | 93-15-2 | 1 | 0 (0, 0) | 0 | 0 (0, 0) |  | X |  |  |  |  |  |
| Azobenzene | 103-33-3 | 1 | 0 (0, 0) | 0 | 0 (0, 0) | X |  | X |  |  |  |  |
| Benzo[a]pyrene | 50-32-8 | 1 | 0 (0, 0) | 0 | 0 (0, 0) |  |  |  |  |  |  | X |
| Citral B | 5392-40-5 | 1 | 0 (0, 0) | 0 | 0 (0, 0) |  | X |  | X |  |  |  |
| Farnesol II | 4602-84-0 | 1 | 0 (0, 0) | 0 | 0 (0, 0) |  | X |  | X |  |  |  |
| Farnesol IV | 4602-84-0 | 1 | 0 (0, 0) | 0 | 0 (0, 0) |  | X |  | X |  |  |  |
| Chrysene | 218-01-9 | 1 | 0 (0, 0) | 0 | 0 (0, 0) |  |  |  |  |  |  | X |
| Fluoranthene | 206-44-0 | 1 | 0 (0, 0) | 0 | 0 (0, 0) |  |  |  |  |  |  | X |
| 1-Naphthylamine | 134-32-7 | 1 | 0 (0, 0) | 0 | 0 (0, 0) | X |  |  |  |  |  |  |
| Simetryn | 1014-70-6 | 1 | 0 (0, 0) | 0 | 0 (0, 0) |  |  | X |  |  |  |  |
| Retene | 483-65-8 | 1 | 0 (0, 0) | 0 | 0 (0, 0) |  |  |  |  |  |  | X |
| 3,5-Dichloroaniline | 626-43-7 | 1 | 0 (0, 0) | 0 | 0 (0, 0) | X |  | X |  |  | X |  |
| Biphenyl | 92-52-4 | 1 | 0 (0, 0) | 0 | 0 (0, 0) | X |  | X |  |  |  |  |
| Terbucarb | 1918-11-2 | 1 | 0 (0, 0) | 0 | 0 (0, 0) |  |  | X |  |  |  |  |
| Bioallethrin | 584-79-2 | 1 | 0 (0, 0) | 0 | 0 (0, 0) |  |  | X |  |  |  |  |
| Tetrachlorvinphos | 961-11-5 | 1 | 0 (0, 0) | 0 | 0 (0, 0) |  |  | X |  |  |  |  |
| D-(cis-trans)-Phenothrin-I | 26002-80-2 | 1 | 0 (0, 0) | 0 | 0 (0, 0) |  |  | X |  |  |  |  |
| 2-Methylphenol | 95-48-7 | 1 | 0 (0, 0) | 0 | 0 (0, 0) | X |  |  |  |  |  |  |
| Cyclopentadecanone | 502-72-7 | 1 | 0 (0, 0) | 0 | 0 (0, 0) |  | X |  |  |  |  |  |
| 3-Nitroaniline | 99-09-2 | 1 | 0 (0, 0) | 0 | 0 (0, 0) | X |  |  |  |  |  |  |
| Prallethrin, cis- | 23031-36-9 | 1 | 0 (0, 0) | 5 | 0 (0, 0) |  |  | X |  |  |  |  |
| Tricresylphosphate, ortho- | 78-30-8 | 1 | 0 (0, 0) | 0 | 0 (0, 0) | X |  |  |  | X |  |  |
| Trans-Nonachlor | 39765-80-5 | 0.4 | 0 (0, 0) | 0 | 0 (0, 0) |  |  | X |  |  |  |  |
| 6-Methylchrysene | 1705-85-7 | 0.4 | 0 (0, 0) | 0 | 0 (0, 0) |  |  |  |  |  |  | X |
| Azoxystrobin | 131860-33-8 | 0.4 | 0 (0, 0) | 5 | 0 (0, 0) |  |  | X |  |  |  |  |
| Cyphenothrin cis- | 39515-40-7 | 0.4 | 0 (0, 0) | 0 | 0 (0, 0) |  |  | X |  |  |  |  |
| Cinerin II | 121-20-0 | 0.4 | 0 (0, 0) | 0 | 0 (0, 0) |  |  | X |  |  |  |  |
| Tetramethrin II | 999050-03-2 | 0.4 | 0 (0, 0) | 0 | 0 (0, 0) |  |  | X |  |  |  |  |
| D-(cis-trans)-Phenothrin-II | 999034-03-6 | 0.4 | 0 (0, 0) | 0 | 0 (0, 0) |  |  | X |  |  |  |  |
| XMC (3,4-Dimethylphenyl N-methylcarbamate) | 2425-10-7 | 0.4 | 0 (0, 0) | 0 | 0 (0, 0) |  |  | X |  |  |  |  |
| 1,8-Dimethylnaphthalene | 569-41-5 | 0.4 | 0 (0, 0) | 0 | 0 (0, 0) |  |  |  |  |  |  | X |
| 1,4-Dimethylnaphthalene | 571-58-4 | 0.4 | 0 (0, 0) | 0 | 0 (0, 0) |  |  |  |  |  |  | X |
| 4-Chlorophenol | 106-48-9 | 0.4 | 0 (0, 0) | 0 | 0 (0, 0) | X |  |  |  |  |  |  |
| Alpha-Chlordane | 5103-71-9 | 0.4 | 0 (0, 0) | 0 | 0 (0, 0) |  |  | X |  |  |  |  |
| Anisyl alcohol | 105-13-5 | 0.4 | 0 (0, 0) | 0 | 0 (0, 0) |  | X |  |  |  |  |  |
| Cinnamyl alcohol | 104-54-1 | 0.4 | 0 (0, 0) | 0 | 0 (0, 0) |  | X |  |  |  |  |  |
| METHYL 2-OCTYNOATE | 111-12-6 | 0.4 | 0 (0, 0) | 0 | 0 (0, 0) |  | X |  | X |  |  |  |
| 2-Chloroaniline | 95-51-2 | 0.4 | 0 (0, 0) | 0 | 0 (0, 0) | X |  |  |  |  |  |  |
| Benzotriazole | 95-14-7 | 0.4 | 0 (0, 0) | 0 | 0 (0, 0) | X |  |  |  |  |  |  |
| 2,6-Diethylaniline | 579-66-8 | 0.4 | 0 (0, 0) | 0 | 0 (0, 0) | X |  |  |  |  |  |  |
| 2,6-Diisopropylaniline | 24544-04-5 | 0.4 | 0 (0, 0) | 0 | 0 (0, 0) | X |  |  |  |  |  |  |
| 2,3,5-Trimethylphenol | 697-82-5 | 0.4 | 0 (0, 0) | 0 | 0 (0, 0) | X | X |  |  |  |  |  |
| 2,6-Dimethoxyphenol | 91-10-1 | 0.4 | 0 (0, 0) | 0 | 0 (0, 0) | X |  |  | X |  |  |  |
| 4-Isopropylphenol | 99-89-8 | 0.4 | 0 (0, 0) | 5 | 0 (0, 0) | X | X |  |  |  |  |  |
| Benz[a]anthracene | 56-55-3 | 0.4 | 0 (0, 0) | 0 | 0 (0, 0) |  |  |  |  |  |  | X |
| 2,4-Di-tert-amylphenol | 120-95-6 | 0.4 | 0 (0, 0) | 0 | 0 (0, 0) | X |  |  | X |  |  |  |
| 2-Aminophenol | 95-55-6 | 0.4 | 0 (0, 0) | 0 | 0 (0, 0) | X |  |  |  |  |  |  |
| Guaiacol | 90-05-1 | 0.4 | 0 (0, 0) | 0 | 0 (0, 0) | X |  |  |  |  |  |  |
| 4-Alpha-cumylphenol | 599-64-4 | 0.4 | 0 (0, 0) | 0 | 0 (0, 0) | X |  |  |  |  |  |  |
| 4-Methoxyphenol | 150-76-5 | 0.4 | 0 (0, 0) | 0 | 0 (0, 0) | X |  |  |  |  | X |  |
| Cycloate | 1134-23-2 | 0.4 | 0 (0, 0) | 0 | 0 (0, 0) |  |  | X |  |  |  |  |
| Benzo[k]fluoranthene | 207-08-9 | 0.4 | 0 (0, 0) | 0 | 0 (0, 0) |  |  |  |  |  |  | X |
| Dibenzo[a,h]anthracene | 53-70-3 | 0.4 | 0 (0, 0) | 0 | 0 (0, 0) |  |  |  |  |  |  | X |
| Gamma-Chlordane | 5103-74-2 | 0.4 | 0 (0, 0) | 0 | 0 (0, 0) |  |  | X |  |  |  |  |
| Malathion | 121-75-5 | 0.4 | 0 (0, 0) | 0 | 0 (0, 0) |  |  | X |  |  | X |  |
| Methomyl | 16752-77-5 | 0.4 | 0 (0, 0) | 0 | 0 (0, 0) |  |  | X |  |  |  |  |
| 1,4-Naphthoquinone | 130-15-4 | 0.4 | 0 (0, 0) | 0 | 0 (0, 0) |  |  |  |  |  |  | X |
| Phenol | 108-95-2 | 0.4 | 0 (0, 0) | 0 | 0 (0, 0) |  |  |  |  |  | X |  |
| Diphenylamine | 122-39-4 | 0.4 | 0 (0, 0) | 0 | 0 (0, 0) |  |  | X |  |  |  |  |
| Bifenthrin | 82657-04-3 | 0.4 | 0 (0, 0) | 0 | 0 (0, 0) |  |  | X |  |  |  |  |
| PCB 138 | 35065-28-2 | 0.4 | 0 (0, 0) | 0 | 0 (0, 0) |  |  |  |  |  |  |  |
| PCB 153 | 35065-27-1 | 0.4 | 0 (0, 0) | 0 | 0 (0, 0) |  |  |  |  |  |  |  |
| 4H-cyclopenta[def]phenanthren-4-one | 5737-13-3 | 0.4 | 0 (0, 0) | 0 | 0 (0, 0) | X |  |  |  |  |  | X |
| 9-Fluorenone | 486-25-9 | 0.4 | 0 (0, 0) | 0 | 0 (0, 0) | X |  |  |  |  |  | X |
| 3,6-Dimethylphenanthrene | 1576-67-6 | 0.4 | 0 (0, 0) | 0 | 0 (0, 0) |  |  |  |  |  |  | X |
| 1,6-Dimethylnaphthalene | 575-43-9 | 0.4 | 0 (0, 0) | 0 | 0 (0, 0) |  |  |  |  |  |  | X |
| 1,2-Dimethylnaphthalene | 573-98-8 | 0.4 | 0 (0, 0) | 0 | 0 (0, 0) |  |  |  |  |  |  | X |
| 2-Methylanthracene | 613-12-7 | 0.4 | 0 (0, 0) | 0 | 0 (0, 0) |  |  |  |  |  |  | X |
| 1-Methylpyrene | 2381-21-7 | 0.4 | 0 (0, 0) | 0 | 0 (0, 0) |  |  |  |  |  |  | X |
| PCB 91 | 68194-05-8 | 0.4 | 0 (0, 0) | 0 | 0 (0, 0) |  |  |  |  |  |  |  |
| PCB 127 | 39635-33-1 | 0.4 | 0 (0, 0) | 0 | 0 (0, 0) |  |  |  |  |  |  |  |
| PCB 141 | 52712-04-6 | 0.4 | 0 (0, 0) | 0 | 0 (0, 0) |  |  |  |  |  |  |  |
| PCB 143 | 68194-15-0 | 0.4 | 0 (0, 0) | 0 | 0 (0, 0) |  |  |  |  |  |  |  |
| 2,4-Dimethylaniline | 95-68-1 | 0.4 | 0 (0, 0) | 0 | 0 (0, 0) | X |  |  |  |  |  |  |
| Di-n-propyl phthalate | 131-16-8 | 0.4 | 0 (0, 0) | 0 | 0 (0, 0) | X |  |  |  |  | X |  |
| Pyrimethanil | 53112-28-0 | 0.4 | 0 (0, 0) | 0 | 0 (0, 0) |  |  | X |  |  |  |  |
| Dimethametryn | 22936-75-0 | 0.4 | 0 (0, 0) | 0 | 0 (0, 0) |  |  | X |  |  |  |  |
| Bioallethrin S-cyclopentenyl isomer | 28434-00-6 | 0.4 | 0 (0, 0) | 0 | 0 (0, 0) |  |  | X |  |  |  |  |
| TCMTB | 21564-17-0 | 0.4 | 0 (0, 0) | 0 | 0 (0, 0) | X |  | X |  |  |  |  |
| Bromoxynil octanoic acid ester | 1689-99-2 | 0.4 | 0 (0, 0) | 0 | 0 (0, 0) |  |  | X |  |  |  |  |
| Tetramethrin I | 7696-12-0 | 0.4 | 0 (0, 0) | 5 | 0 (0, 0) |  |  | X |  |  |  |  |
| Tetrahydrophthalimide, cis-1,2,3,6- | 27813-21-4 | 0.4 | 0 (0, 0) | 0 | 0 (0, 0) |  |  | X |  |  |  |  |
| TCEP | 115-96-8 | 0.4 | 0 (0, 0) | 0 | 0 (0, 0) |  |  |  |  | X |  |  |
| Musk Moskene | 116-66-5 | 0.4 | 0 (0, 0) | 0 | 0 (0, 0) |  | X |  |  |  |  |  |
| Dipropyl isocinchomeronate | 136-45-8 | 0.4 | 0 (0, 0) | 0 | 0 (0, 0) |  |  | X |  |  |  |  |
| Prothioconazole-desthio | 999007-03-3 | 0.4 | 0 (0, 0) | 0 | 0 (0, 0) |  |  | X |  |  |  |  |
| Chlorfenapyr | 122453-73-0 | 0.4 | 0 (0, 0) | 0 | 0 (0, 0) |  |  | X |  |  |  |  |
| Trifenmorph | 1420-06-0 | 0.4 | 0 (0, 0) | 0 | 0 (0, 0) |  |  | X |  |  |  |  |

| Supplemental Table 2. Summary of chemicals detected in silicone wristbands by MyExposome classification. | | | | |
| --- | --- | --- | --- | --- |
|  | 12 Gestational Weeks | | 24 Gestational Weeks | |
|  | (n = 255) | | (n = 20) | |
| MyExposome Classification | Median (IQR) | Range | Median (IQR) | Range |
| Total | 23 (20, 26) | (12, 37) | 19 (18, 22) | (16, 28) |
| Chemicals in commerce | 12 (10, 13) | (6, 20) | 10 (9, 12) | (7, 14) |
| Personal care products | 10 (8, 11) | (3, 19) | 9 (7, 9) | (5, 13) |
| Pesticides | 6 (5, 8) | (2, 15) | 7 (5, 7) | (4, 11) |
| Consumer products | 2 (1, 3) | (0, 8) | 2 (1, 2) | (0, 3) |
| Flame retardants | 1 (1, 2) | (0, 4) | 1 (1, 2) | (0, 3) |
| Pharmacological | 1 (1, 2) | (0, 4) | 1 (0, 1) | (0, 2) |
| PAHs | 1 (0, 2) | (0, 10) | 0 (0, 1) | (0, 1) |
| Note: Chemicals may be classified within more than one category. See Supplemental Table 1 for chemical classifications.  Abbreviations: IQR, interquartile range; PAHs, polycyclic aromatic hydrocarbons. | | | | |

| Supplemental Table 3. Multivariable linear regression of specific characteristics in relation to wristband chemical detection or chemical concentrations. | | |
| --- | --- | --- |
| Characteristic | Chemical | Beta (95% CI) |
| Nail polish use^a^ | Number of detected personal care product chemicals | 0.43 (-0.22, 1.09) |
|  | Triphenyl phosphate | 0.27 (0.00, 0.55) |
|  | Di-n-butyl phthalate | -0.10 (-0.37, 0.17) |
|  | Diisobutyl phthalate | -0.21 (-0.49, 0.07) |
|  | Butyl benzyl phthalate | -0.02 (-0.31, 0.27) |
|  | Di-n-nonyl phthalate | -0.09 (-0.39, 0.20) |
|  | Diethyl phthalate | 0.01 (-0.28, 0.30) |
| Handwashing^b^ | Number of detected chemicals | -0.02 (-0.11, 0.07) |
| Gardening^c^ | Number of detected pesticides | 0.28 (-0.34, 0.91) |
|  | Di-n-butyl phthalate | 0.18 (-0.14, 0.50) |
|  | N,N-Diethyl-m-toluamide | 0.16 (-0.14, 0.47) |
|  | Benzyl benzoate | -0.07 (-0.37, 0.24) |
|  | Permethrin | 0.11 (-0.26, 0.49) |
|  | Diethyl phthalate | 0.01 (-0.31, 0.34) |
| Parity^d^ | Triphenyl phosphate | -0.07 (-0.35, 0.20) |
| Season - Fall^e^ | Benzophenone | Ref |
| Season - Winter^e^ |  | -0.02 (-0.39, 0.35) |
| Season - Spring^e^ |  | 0.05 (-0.30, 0.40) |
| Season - Summer^e^ |  | 0.23 (-0.18, 0.65) |
| Season - Fall^e^ | Number of detected pesticides | Ref |
| Season - Winter^e^ |  | -0.82 (-1.51, -0.13) |
| Season - Spring^e^ |  | -0.15 (-0.81, 0.51) |
| Season - Summer^e^ |  | -0.13 (-0.89, 0.63) |
| Note: Multivariable linear regression models adjusted for age (linear), body mass index (BMI, linear), educational attainment (less than college graduate, college graduate, any post-graduate), marital status (married, unmarried), race and ethnicity (White Non-Hispanic, other), parity (0, ≥1), self-reported smoke exposure during pregnancy (first- or second-hand exposure, none), season (winter, spring, fall, summer), and gestational age the wristband was first worn (linear). Chemical concentrations median standardized to batch, and centered (mean = 0) and scaled (standard deviation = 1). Missing covariate data imputed via multiple imputation. ^a^Reported first trimester nail polish use (hand or foot); referent is no reported use relative to any reported use. ^b^Reported average number of times handwashing per day; linear.  ^c^Reported gardening in first trimester; referent is no reported gardening in first trimester relative to reported gardening in first trimester.  ^d^Parous versus nulliparous women.  ^e^Season of wear for the wristband; referent is fall, relative to winter, spring, and summer. | | |

| Supplemental Table 4. Multivariable linear regression of season^a^ in relation to chemical concentrations measured in wristbands worn at 12 gestational weeks. | | | |
| --- | --- | --- | --- |
| Chemical | Spring | Summer | Winter |
| Di-n-butyl phthalate | -0.10 (-0.44, 0.24) | -0.10 (-0.50, 0.29) | -0.10 (-0.46, 0.26) |
| Galaxolide | 0.10 (-0.24, 0.45) | -0.12 (-0.52, 0.28) | -0.04 (-0.40, 0.32) |
| Diisobutyl phthalate | 0.17 (-0.17, 0.51) | -0.03 (-0.43, 0.37) | -0.27 (-0.63, 0.09) |
| Butyl benzyl phthalate | 0.33 (-0.01, 0.68) | 0.09 (-0.31, 0.49) | 0.07 (-0.29, 0.43) |
| Lilial | 0.14 (-0.20, 0.49) | 0.04 (-0.36, 0.44) | 0.05 (-0.31, 0.41) |
| Benzyl salicylate | 0.42 (0.08, 0.75) | 0.05 (-0.35, 0.44) | -0.01 (-0.37, 0.34) |
| Tonalide | 0.22 (-0.13, 0.56) | 0.13 (-0.27, 0.53) | 0.07 (-0.29, 0.43) |
| N,N-Diethyl-m-toluamide | 0.09 (-0.25, 0.42) | 0.27 (-0.12, 0.66) | -0.17 (-0.52, 0.19) |
| Benzophenone | 0.05 (-0.30, 0.40) | 0.23 (-0.18, 0.65) | -0.02 (-0.39, 0.35) |
| Ethylene brassylate | 0.10 (-0.24, 0.43) | -0.15 (-0.53, 0.24) | 0.42 (0.06, 0.78) |
| Benzyl benzoate | -0.16 (-0.50, 0.17) | -0.30 (-0.70, 0.10) | -0.14 (-0.49, 0.21) |
| Di-n-nonyl phthalate | -0.01 (-0.35, 0.33) | 0.18 (-0.21, 0.57) | 0.10 (-0.26, 0.46) |
| Permethrin | -0.04 (-0.39, 0.31) | 0.21 (-0.19, 0.61) | -0.15 (-0.51, 0.21) |
| Diethyl phthalate | 0.35 (0.00, 0.69) | 0.06 (-0.34, 0.45) | 0.27 (-0.09, 0.64) |
| Butylated hydroxyanisole | -0.29 (-0.63, 0.05) | -0.37 (-0.76, 0.03) | 0.03 (-0.33, 0.39) |
| 2,4-Di-tert-butylphenol | 0.45 (0.12, 0.79) | 0.00 (-0.38, 0.38) | -0.04 (-0.39, 0.31) |
| Note: Multivariable linear regression models adjusted for age (linear), body mass index (BMI, linear), educational attainment (less than college graduate, college graduate, any post-graduate), marital status (married, unmarried), race and ethnicity (White Non-Hispanic, other), parity (0, ≥1), self-reported smoke exposure during pregnancy (first- or second-hand exposure, none), and gestational age the wristband was first worn (linear). Chemical concentrations median standardized to batch, and centered (mean = 0) and scaled (standard deviation = 1). Missing covariate data imputed via multiple imputation. ^a^Season of wear for the wristband; referent is fall, relative to winter, spring, and summer. | | | |

| Supplemental Table 5. Median concentrations of chemicals measured in wristbands worn at 12 gestational weeks by SOM profile. | | | | | | |
| --- | --- | --- | --- | --- | --- | --- |
|  |  | Profile | | | | |
| Chemical | Primary Class | 1  (n = 15) | 2 (n = 149) | 4 (n = 19) | 5 (n = 34) | 7 (n = 10) |
| Butyl benzyl phthalate | Phthalate | -0.42 | -0.38 | 2.37 | -0.37 | -0.10 |
| Di-n-butyl phthalate | Phthalate | -0.16 | -0.21 | 0.32 | -0.42 | 2.41 |
| Di-n-nonyl phthalate | Phthalate | -0.34 | -0.34 | -0.31 | -0.12 | -0.34 |
| Diethyl phthalate | Phthalate | -0.02 | -0.24 | -0.24 | -0.10 | -0.34 |
| Diisobutyl phthalate | Phthalate | -0.25 | -0.31 | 0.05 | -0.14 | 3.25 |
| Benzyl benzoate | Pesticide | -0.33 | -0.30 | -0.31 | 0.42 | -0.15 |
| N,N-Diethyl-m-toluamide | Pesticide | -0.25 | -0.23 | -0.17 | -0.22 | -0.19 |
| Permethrin | Pesticide | -0.19 | -0.21 | -0.15 | -0.23 | -0.22 |
| 2,4-Di-tert-butylphenol | Chemicals in commerce | 1.80 | -0.24 | -0.24 | -0.19 | 0.42 |
| Galaxolide | Chemicals in commerce | -0.32 | -0.44 | -0.34 | 1.79 | -0.53 |
| Benzophenone | Personal care products | 0.09 | -0.15 | -0.21 | -0.09 | 0.26 |
| Benzyl salicylate | Personal care products | -0.48 | -0.30 | -0.17 | 0.49 | -0.30 |
| Butylated hydroxyanisole | Personal care products | -0.18 | -0.18 | 0.06 | -0.20 | -0.13 |
| Ethylene brassylate | Personal care products | -0.29 | -0.33 | -0.19 | -0.09 | -0.51 |
| Lilial | Personal care products | -0.32 | -0.36 | -0.27 | 0.14 | -0.50 |
| Tonalide | Personal care products | -0.17 | -0.27 | -0.23 | -0.09 | -0.29 |
| Chemical concentrations median standardized to batch, and centered (mean = 0) and scaled (standard deviation = 1). | | | | | | |
